# MAM‐Localized MANF Counteracts Microinflammatory Stress to Attenuate Mitochondrial Dysfunction and Cataractogenesis in High Myopia

**DOI:** 10.1002/advs.76342

**Published:** 2026-07-08

**Authors:** Xin Liu, Hao Li, Ching Kang, Dongling Guo, Shuyu Liu, Jiaqi Meng, Zhiqian Huang, Yu Du, Xiangjia Zhu

**Affiliations:** ^1^ Department of Ophthalmology Eye & ENT Hospital Fudan University Shanghai China; ^2^ Key Laboratory of Myopia and Related Eye Diseases NHC Key Laboratory of Myopia and Related Eye Diseases Chinese Academy of Medical Sciences Shanghai China; ^3^ Shanghai Key Laboratory of Visual Impairment and Restoration Shanghai China; ^4^ State Key Laboratory of Brain Function and Disorders Shanghai China; ^5^ Department of Ophthalmology Zhongshan Hospital Affiliated to Fudan University Shanghai China

**Keywords:** highly myopic cataract, MANF, microinflammation, mitochondria‐associated ER membranes (MAMs), SERCA2

## Abstract

Chronic microinflammation drives tissue degeneration, particularly in age‐related and metabolic diseases, yet how it disrupts inter‐organelle communication that leads to cellular failure remains largely unexplored. Utilizing highly myopic cataract (HMC) as a paradigm, we uncover a non‐canonical defense mechanism centered on mitochondria‐associated endoplasmic reticulum membranes (MAMs). Under microinflammatory stress, mesencephalic astrocyte‐derived neurotrophic factor (MANF), conventionally recognized as an ER‐resident protein, specifically localizes to MAMs in lens epithelial cells (LECs). At this critical interface, MANF acts as a metabolic sensor that safeguards calcium homeostasis by directly promoting the ubiquitin‐mediated degradation of the sarco/endoplasmic reticulum Ca^2^^+^‐ATPase 2 (SERCA2). Microinflammation‐induced MANF deficiency triggers pathological SERCA2 accumulation, MAM hyperassembly, disrupted ER‐to‐mitochondria calcium coupling, profound oxidative stress, and mitochondrial bioenergetic collapse, culminating in LEC apoptosis. We validate this pathogenic cascade using human HMC specimens, a unilateral defocus‐induced high myopia model, and a novel lens‐specific Manf conditional knockdown mouse. Strikingly, in vivo AAV2‐mediated MANF gene delivery successfully normalizes MAM architecture, rescues mitochondrial function, and prevents cataractogenesis, demonstrating therapeutic reversibility. In summary, this study establishes the MANF‐SERCA2 axis at the MAM interface as a critical pathway linking microinflammation to organelle dysfunction and proposes this interaction as a promising therapeutic target for cataractogenesis and other microinflammation‐driven degenerations.

## Graphical abstract

Chronic microinflammation in high myopia suppresses MANF in lens epithelial cells. MANF normally resides within MAMs and promotes ubiquitin‐mediated degradation of the ER Ca^2^^+^ pump SERCA2. MANF loss causes pathological SERCA2 accumulation, MAM hyperassembly, and disrupted ER‐to‐mitochondria Ca^2^^+^ transfer, leading to mitochondrial failure, oxidative stress, and apoptosis, thereby accelerating cataract formation.

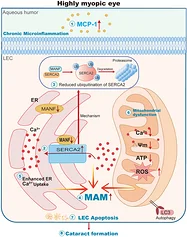

## 1 Introduction

Chronic, low‐grade microinflammation is increasingly recognized as a fundamental pathogenic driver in numerous age‐related and metabolic diseases [1, 2]. Yet, despite decades of research on inflammatory cytokines and oxidative stress, a critical mechanistic gap remains: how is sustained extracellular microinflammatory signaling fundamentally transduced into intracellular organelle failure? This question is particularly pertinent because the early stages of tissue degeneration—prior to overt apoptosis or autophagy exhaustion—are characterized by subtle but progressive disruptions in inter‐organelle communication [1, 2, 3, 4, 5, 6]. Mitochondria, as the metabolic powerhouses of the cell, depend critically on their physical and functional tethering with the endoplasmic reticulum (ER) at specialized contact sites termed mitochondria‐associated ER membranes (MAMs) [6, 7, 8]. These dynamic signaling hubs orchestrate ER‐to‐mitochondria calcium (Ca^2^^+^) transfer, lipid metabolism, and autophagic flux—processes essential for cellular homeostasis and survival [8, 9]. While emerging evidence suggests that MAM integrity is exquisitely sensitive to inflammatory stress, the precise molecular sensors and effectors that transduce extracellular microinflammatory signals into MAM structural remodeling remain largely uncharacterized [10, 11].

Highly myopic cataract (HMC) serves as an unprecedented clinical and biological paradigm to interrogate these microinflammation‐driven mechanisms [12, 13]. Unlike common age‐related cataracts, HMC develops prematurely in working‐age individuals, striking 10–20 years earlier, and is driven by a distinctive ocular microenvironment characterized by the persistent elevation of pro‐inflammatory cytokines [14, 15, 16, 17, 18]. In particular, monocyte chemoattractant protein‐1 (MCP‐1/CCL2) is consistently upregulated in the highly myopic eye [19, 20, 21], establishing a chronic state of local microinflammation. This sustained inflammatory milieu imposes an extraordinary metabolic and redox burden on long‐lived lens epithelial cells (LECs), which must maintain protein homeostasis and mitochondrial function across decades without cell division [22, 23, 24, 25]. Consequently, LECs in the HM microenvironment exhibit heightened oxidative stress, accelerated senescence, and prematurely triggered apoptosis [26, 27, 28, 29, 30]. While previous studies have documented oxidative damage and dysregulated autophagy in HMC [31, 32, 33], the upstream initiating event—specifically, whether extracellular MCP‐1 directly disrupts ER‐mitochondrial tethering and inter‐organelle Ca^2^^+^ coupling—has not been systematically investigated.

Mesencephalic astrocyte‐derived neurotrophic factor (MANF) is an evolutionarily conserved secreted protein traditionally characterized as an ER‐resident cytoprotective factor [34, 35]. Although recent studies have expanded its known functions to include mitigating neuroinflammation and preserving mitochondrial homeostasis, proteomic studies have hinted at its potential enrichment at MAM‐associated protein complexes [3, 6, 34, 35, 36]. However, this observation has remained largely unexplored. A critical knowledge gap thus persists: Does MANF dynamically stabilize MAM architecture under microinflammatory stress, and if so, through what molecular mechanism? Furthermore, which downstream effector proteins at MAMs are regulated by MANF to preserve ER‐mitochondria Ca^2^^+^ coupling? [37, 38, 39, 40] Notably, the sarco/endoplasmic reticulum Ca^2^^+^‐ATPase 2 (SERCA2) is a central regulator of ER Ca^2^^+^ refilling and ER‐to‐mitochondria Ca^2^^+^ transfer [41, 42, 43, 44, 45]. Whether MANF governs SERCA2 stability through post‐translational modifications—particularly ubiquitin‐mediated proteasomal degradation—and whether this protective mechanism is compromised under myopic microinflammatory stress, remains entirely unknown.

Here, we test the central hypothesis that MAM‐localized MANF protects against cataractogenesis in high myopia by serving as a luminal quality‐control sensor that promotes the ubiquitin‐mediated degradation of SERCA2, thereby maintaining optimal ER‐mitochondria Ca2^+^ coupling under microinflammatory stress. Utilizing a comprehensive platform encompassing clinical HMC specimens, mouse models, and cellular assays, we demonstrate that MCP‐1‐driven microinflammation fundamentally disrupts MAM architecture by suppressing MANF. Mechanistically, we reveal that intraluminal MANF regulates the transmembrane SERCA2 pump, and its deficiency triggers pathological SERCA2 accumulation, accelerated Ca^2+^ reuptake, and terminal mitochondrial bioenergetic collapse. Importantly, restoring this regulatory axis via in vivo gene therapy successfully normalizes MAM integrity and attenuates accelerated cataractogenesis.

## 2 Results

### 2.1 Consistent MANF Downregulation in Lens From Clinical HMC to Experimental Myopic Microinflammation

In contrast to the peripheral cortical opacities typically seen in ARC, HMC presents early‐onset nuclear sclerosis (Figure 1a) and a distinct microenvironment of microinflammation characterized by elevated MCP‐1 in aqueous humor (Figure 1b). Using lens explants from unilateral defocus‐induced HM mice (Figure 1c,d), MCP‐1 was proven to significantly aggravate the lens opacification in HM eyes in contrast to the contralateral control eyes (Figure 1e,f).

**FIGURE 1:**
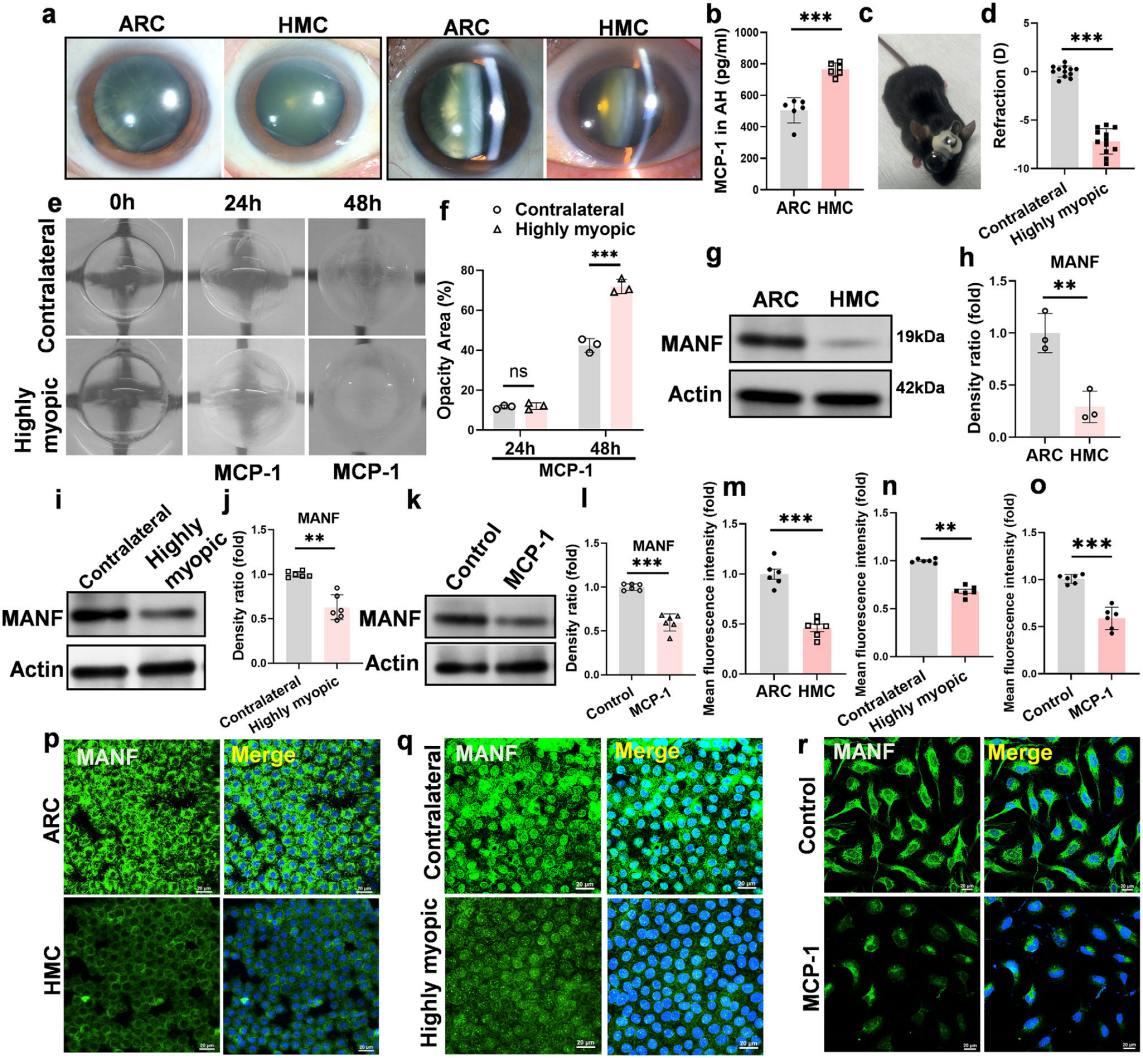
Reduced MANF expression in the lens of HMC patients and mimetic models. (a) Representative slit‐lamp photographs of typical age‐related cataract (ARC, peripheral cortical opacities) and highly myopic cataract (HMC, dense nuclear opacity). (b) Aqueous humor (AH) levels of monocyte chemoattractant protein‐1 (MCP‐1) detected by enzyme‐linked immunosorbent assay (ELISA) (*n* = 6). (c) Representative photograph of a mouse with defocus‐induced high myopia (HM) in the right eye, wearing a ‐10 D lens. (d) Refraction measurements of the contralateral and the defocused HM eyes of the mice (*n* = 12). (e,f) Representative images and quantification of lens opacity from HM and the contralateral eyes of mice before and after 100 nM MCP‐1 treatment. The opacity area (%) is defined as the ratio of the projection area of opacification to that of the whole lens (*n* = 3). (g,h,m,p) MANF protein expression in lens epithelia from HMC and ARC patients (Western blot *n* = 3, immunofluorescence staining *n* = 6). (i,j,n,q) MANF protein expression in lens epithelia from the contralateral and HM eyes of mice (Western blot *n* = 6, immunofluorescence staining *n* = 6). (k,l,o,r) MANF protein expression in human SRA 01/04 lens epithelial cells (LECs) exposed to 100 nM MCP‐1 (48 h) (Western blot *n* = 6, immunofluorescence staining *n* = 6). Data are presented as mean ± standard deviation (SD). Significance was determined using unpaired t‐tests. n = number of biological replicates; ns = not significant; ^*^ *p* < 0.05, ^**^ *p* < 0.01, ^***^ *p* < 0.001.

To investigate whether MANF participates in HMC pathogenesis, we first evaluated its expression in clinical lens epithelia. MANF was found significantly downregulated in HMC patients compared with ARC controls at both mRNA and protein levels (Figure 1g,h,m,p and Figure S1a). This reduction was consistently observed in lens epithelia from the defocused eyes of the unilateral HM mice, compared with their contralateral control eyes (Figure 1i,j,n,q).

Furthermore, to test whether microinflammatory stimuli drive MANF suppression in the lens, we exposed human LECs (SRA 01/04) to MCP‐1—the key proinflammatory biomarker elevated in HM eyes. Within 48 h, MANF expression was markedly reduced (Figure 1k,l,o,r). Together, these findings indicate that MANF downregulation is a consistent feature of lens in response to myopic microinflammatory stimuli across human, mouse, and cellular contexts.

### 2.2 Deficiency of MAM‐Localized MANF Leads to Hyperassembly of MAM in HMC

Previous studies have established diverse functional roles for MANF across various cellular compartments. To elucidate the impact of MANF on cataractogenesis in HM eyes, we first sought to characterize its subcellular localization in human LECs. To avoid optical superposition and species cross‐reactivity, we utilized multiplexed high‐resolution immunofluorescence (Figure 2a–d). This approach clearly distinguished a dual‐distribution pattern for MANF: while a large luminal pool broadly colocalized with the diffuse ER network (Calnexin), a specific subset of MANF displayed distinct, punctate enrichments selectively docking at the boundaries of the mitochondrial network (labeled by either MitoTracker or TOMM20). These precise tri‐localization interfaces structurally confirm that MANF is specifically enriched at ER‐mitochondria contact sites (MAMs). Subcellular fractionation followed by western blot analysis further substantiated this finding. MANF was enriched in both the ER fraction (marked by inositol 1,4,5‐trisphosphate receptor type 1, IP3R1 and SERCA2) and the MAM fraction (marked by acyl‐CoA synthetase long‐chain family member 4, ACSL4), with the highest abundance detected in the ER, followed closely by the MAM compartment (Figure 2f, left panels). To precisely define the topological orientation of MANF within the MAM architecture, we performed a Proteinase K protection assay on purified MAM vesicles. In intact MAM vesicles, MANF was completely protected from Proteinase K digestion, behaving identically to the canonical ER‐luminal chaperone Glucose‐Regulated Protein 78 / Binding immunoglobulin Protein (GRP78/BiP). In stark contrast, the outer‐mitochondrial membrane marker Translocase of Outer Mitochondrial Membrane 20 (TOMM20) and the exposed cytosolic domains of Voltage‐Dependent Anion Channel (VDAC) were readily degraded. MANF was only degraded when membrane integrity was disrupted by Triton X‐100 permeabilization (Figure 2f, right panels). These results demonstrate that MANF resides inside the ER/MAM lumen, not on the cytosolic face or as a transmembrane protein. Furthermore, co‐immunoprecipitation (Co‐IP) assays confirmed a robust interaction between MANF and KDEL receptor (KDELR2, Figure S3a). Given that KDELRs selectively capture and retrieve ER luminal proteins, this interaction—mediated by MANF's C‐terminal RTDL motif—provides the molecular basis for its steady‐state retention within the ER network. To precisely define MANF's localization at organelle contact sites, we employed a split green fluorescent protein (spGFP) assay specific for MAMs. This assay demonstrated significant enrichment of MANF at spGFP labeled MAMs, indicating its expression at ER‐mitochondria contact sites (Figure 2e).

**FIGURE 2:**
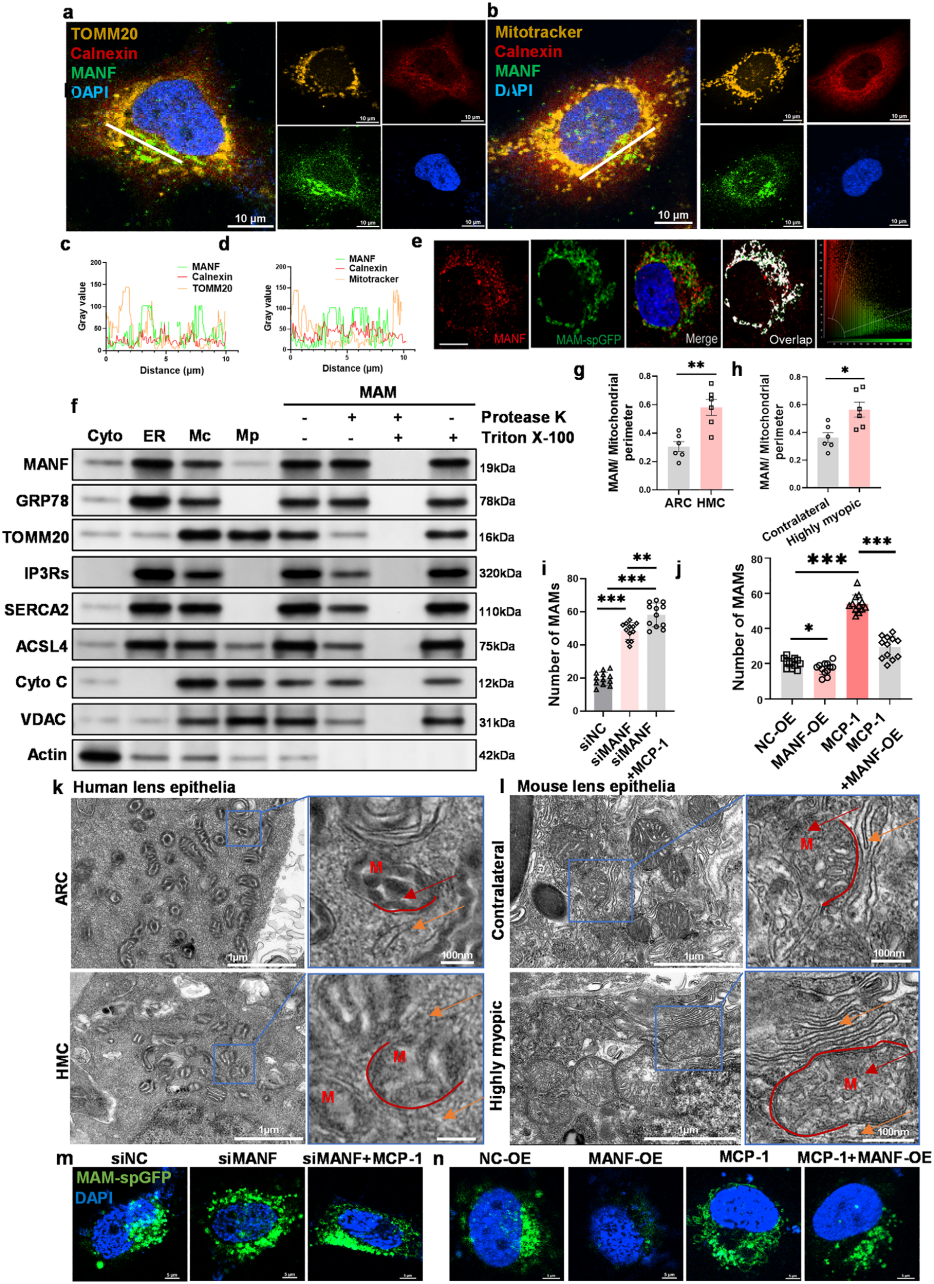
MANF deficiency leads to MAM hyperassembly in HMC. (a,b) Immunofluorescence (IF) analysis of the spatial relationship among MANF, mitochondria, and the endoplasmic reticulum (ER) in SRA 01/04 human lens epithelial cells (LECs). Cells were stained with MitoTracker or anti‐TOMM20 (translocase of outer mitochondrial membrane 20, for mitochondria, yellow), anti‐Calnexin (ER, red), and anti‐MANF (green). Nuclei were counterstained with DAPI (blue). Scale bars: 10 µm. (c,d) Line profiles (white lines in a and b) were analyzed using Image J software to quantify MANF fluorescence intensity and its co‐localization with mitochondrial and ER markers. (e) IF images showing MANF (red) co‐localization with split green fluorescent protein (spGFP) expressed MAMs (green). Nuclei were stained with DAPI (blue). White areas in overlay indicate regions of co‐localization between MANF and spGFP signals. The scatter plot illustrates the degree of co‐localization. Scale bars: 5 µm. (f) Western blot analysis of MANF subcellular localization (left) and its topology at the MAMs (right). *Left panels*: Immunoblotting of MANF and specific organelle markers in different subcellular fractions isolated from SRA 01/04 LECs via density‐gradient centrifugation. Inositol 1,4,5‐trisphosphate receptor type 1 (IP3R1) and sarco/endoplasmic reticulum Ca^2^^+^‐ATPase 2b (SERCA2b) indicate the ER; acyl‐CoA synthetase long‐chain family member 4 (ACSL4) indicates MAMs; cytochrome C (Cyto C) indicates mitochondria; voltage‐dependent anion channel (VDAC) indicates MAMs and mitochondria. Cyto, cytoplasm; Mc, crude mitochondrial fraction; Mp, purified mitochondrial fraction. *Right panels*: Proteinase K (PK) protection assay on the isolated MAM fraction, analyzed by Western blot. Intact MAMs were subjected to PK digestion in the presence or absence of the membrane‐permeabilizing detergent Triton X‐100 prior to immunoblotting. Glucose‐Regulated Protein 78 / Binding immunoglobulin Protein (GRP78/ BiP) serves as an ER‐luminal protected control, while TOMM20 and VDAC serve as outer membrane‐exposed controls. (g,h,k,l) Representative transmission electron microscopy (TEM) images from human (g,k) and mouse (h,l) lens epithelia showing MAM, with quantitative measurements of MAM lengths (*n* = 6). The red lines denote the MAM length, defined as the closely apposed regions between mitochondria (M; red arrows) and ER (orange arrows). Quantification represents the MAM length as a percentage of the total mitochondrial perimeter. Blue rectangles outline the areas shown in the enlarged views. Scale bars: 1 µm (overview), 100 nm (enlargements). (i,j,m,n) IF images of spGFP MAMs reporter (green) and quantification of MAM puncta (n = 12) in SRA01/04 LECs subjected to MANF modulation and MCP‐1 intervention. Scale bars: 5 µm. Data are presented as mean ± SD. Level of significance was detected using unpaired t‐test (g,h) and two‐sided one‐way ANOVA with Tukey's multiple comparisons test (i,j). n represents biological replicates; ^*^ *p* < 0.05, ^**^ *p* < 0.01, ^***^ *p* < 0.001.

We next investigated the possible pathological role of MANF at MAMs in HMC. Transmission electron microscopy (TEM) analysis of human lens epithelia first identified that the MAM length was 1.91‐fold greater in HMC compared with ARC controls (Figure 2g,k), a trend also observed in the mouse lens epithelia of HM eyes compared to their contralateral controls (1.55‐fold increase, Figure 2h,l). To determine whether MANF directly regulates MAM assembly, we modulated its expression in SRA 01/04 LECs. siRNA‐mediated knockdown of MANF (resulting in a 94.4% reduction in mRNA and an 82.34% reduction in protein; Figure S1b–d) was sufficient to induce excessive MAM formation (Figure S1h,i), recapitulating the MAM hyperassembly phenotype observed in HMC.

We next employed a spGFP based MAM reporter to quantitatively assess ER–mitochondria contact sites. Knockdown of MANF significantly increased the number of MAM puncta (Figure 2i,m and Figure S1j), and this effect was further augmented upon intervention with the pro‐inflammatory cytokine MCP‐1 (Figure 2i,m). Conversely, lentiviral overexpression of MANF (achieving a 4.69‐fold increase in mRNA and a 4.13‐fold increase in protein; Figure S1e–g) not only reduced basal MAM contacts but also, with a more pronounced effect, reversed the excessive increase in MAM puncta triggered by MCP‐1 (Figure 2j,n and Figure S1j).

Taken together, these findings establish MANF as a MAM‐resident protein in human LECs and highlight the pivotal role of MANF loss in driving pathological MAM remodeling under myopic microinflammatory stress. This directly links reduced MANF expression to aberrant ER–mitochondria coupling in HMC pathogenesis.

### 2.3 Hyperassembly of MAM Initiates a Pathogenic Cascade From Calcium Mishandling to Apoptosis in LECs

Given the established role of MAMs as hubs for ER–mitochondria Ca^2^^+^ transfer, we investigated whether MANF depletion perturbs cellular Ca^2^^+^ dynamics in SRA 01/04 LECs. Upon ATP stimulation, control LECs exhibited a sharp rise in cytosolic Ca^2+^, reflected by a rapid increase in Fluo‐4 fluorescence intensity ；‐ however ， this response was markedly blunted by MANF knockdown (Figure 3a, left). Concurrently, mitochondrial Ca^2^^+^ uptake, measured by Rhod‐2 fluorescence, was also reduced (Figure 3a, right). Consistent with this impaired Ca^2^^+^ flux, protein levels of key Ca^2^^+^ handling components at the ER–mitochondria interface—including the inositol 1,4,5‐trisphosphate receptor (IP3R; an ER Ca^2^^+^ release channel), the voltage‐dependent anion channel (VDAC; a mitochondrial outer membrane porin), and the mitochondrial calcium uniporter (MCU; the inner membrane Ca^2^^+^ uptake protein)—were all downregulated following MANF knockdown (Figure S2a,b) and were all restored by MANF overexpression in MCP‐1 treated LECs (Figure S2c,d).

**FIGURE 3:**
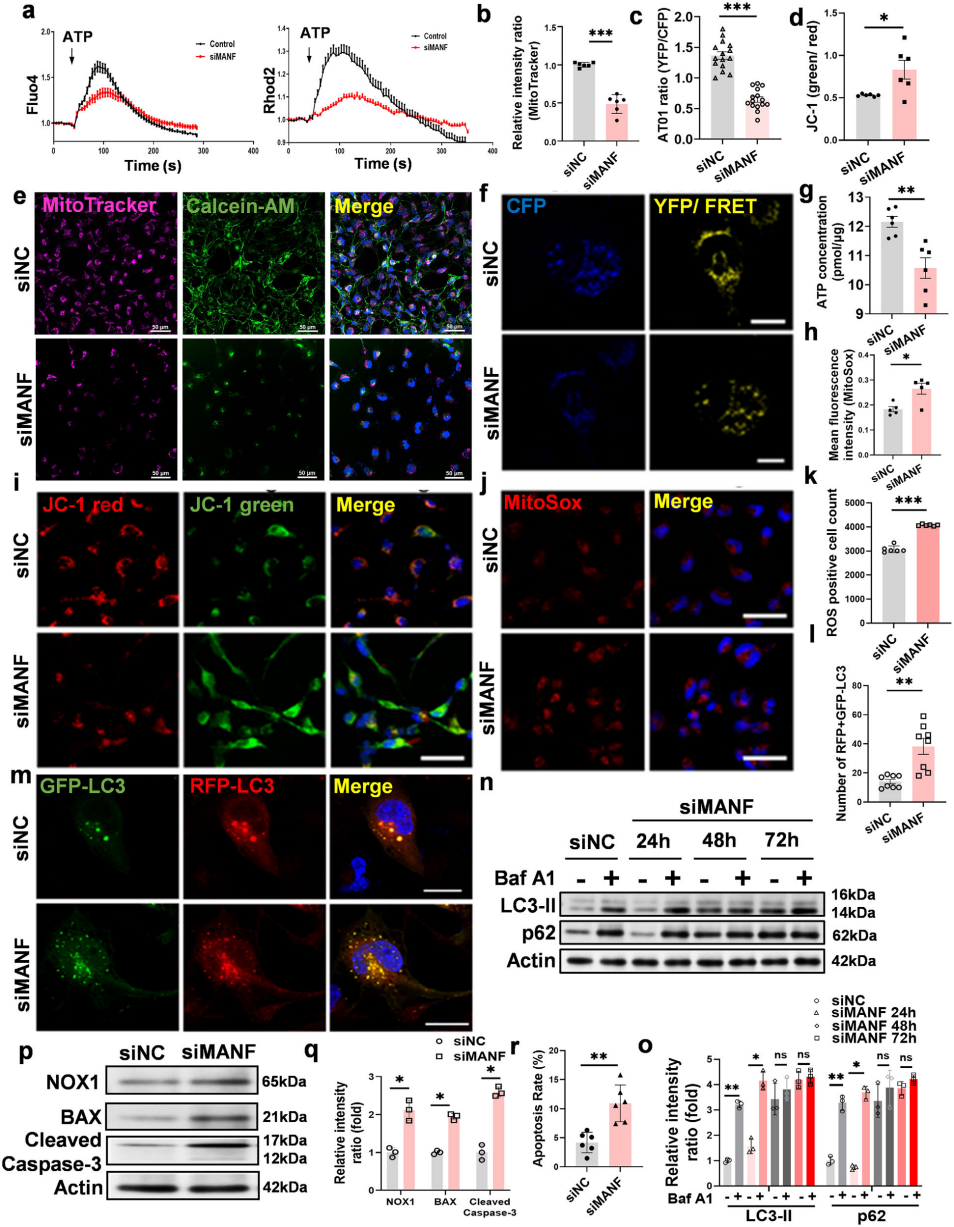
MANF deficiency in LECs is followed by disturbance of Ca^2+^ transfer and mitochondrial bioenergetics, and ultimately apoptosis. (a) Measurement of cytosolic and mitochondrial Ca^2^^+^ fluxes in SRA 01/04 human lens epithelial cells (LECs) following ATP stimulation (*n* = 3). The fluorescence intensities of cytosolic and mitochondrial Ca^2^^+^ were monitored using Fluo‐4 and Rhod‐2 probes, respectively. (b–f,i) Changes of mitochondrial membrane potential (ΔΨm) in SRA 01/04 LECs after MANF knockdown assessed by multiple independent methods: (b,e) MitoTracker (magenta) and Calcein‐AM (green) co‐staining (*n* = 6), Scale bars: 50 µm; (c,f) FRET‐based mitochondrial membrane potential assay (*n* = 15), Scale bars: 10 µm; (d,i) JC‐1 staining (with mitochondrial depolarization indicated by an increased green/red fluorescence ratio, *n* = 6), Scale bars: 50 µm. (g) Changes of cellular ATP content after MANF knockdown (*n* = 6). (h,j) Changes of mitochondrial superoxide production after MANF knockdown detected by MitoSOX staining (*n* = 5, scale bars: 50 µm). (k) Total cellular reactive oxygen species (ROS) levels measured by flow cytometry (*n* = 6). (l,m) Analysis of autophagic flux in SRA 01/04 LECs using the red fluorescent protein (RFP)‐green fluorescent protein (GFP)‐microtubule‐associated proteins 1A/1B light chain 3B (LC3) reporter. Autophagic flux was quantified by counting autolysosomes (RFP+GFP‐ puncta). The increase in red‐only puncta reflects enhanced autophagic flux. Quantification of autolysosomes counted in non‐adjacent cells per group (*n* = 8). Scale bar: 10 µm. (n,o) Western blot analysis of LC3‐II (a marker for autophagosome formation) and p62 (a substrate degraded by autophagy) in LECs transfected with siMANF for 24, 48, and 72 h. Bafilomycin (Baf) A1 (100 nm) was used to inhibit autophagosome‐lysosome fusion (*n* = 3). (p,q) Protein expression of apoptosis‐related markers NADPH oxidase 1 (NOX1), BCL2‐associated X protein (BAX), and cleaved Caspase‐3 (*n* = 3). (r) Apoptosis rate measured by flow cytometry using fluorescein isothiocyanate (FITC) Annexin V staining (*n* = 6). All data are presented as mean ± SD. Statistical significance was determined using an unpaired t‐test (b,c,d,g,h,k,l,r), two‐sided one‐way ANOVA with Tukey's multiple comparisons test (o) or two‐sided two‐way ANOVA with Šídák’ s multiple comparisons test (q). n represents biological replicates. ^*^ *p* < 0.05, ^**^ *p* < 0.01, ^***^ *p* < 0.001.

Considering that Ca^2^^+^ mishandling may impair mitochondrial bioenergetics, we next assessed the functional consequence of MANF depletion in LECs and observed widespread mitochondrial dysfunction characterized by the collapse in mitochondrial membrane potential (ΔΨm), as shown by MitoTracker staining (Figure 3b,e), a FRET‐based assay (Figure 3c,f), and JC‐1 staining (Figure 3d,i). This was accompanied by a significant decrease in cellular ATP levels (Figure 3g), a marked increase in both mitochondrial superoxide (MitoSOX Red, Figure 3h,j) and overall cellular ROS production (Figure 3k and Figure S2e). Furthermore, this oxidative burden compromised local redox homeostasis, as featured by downregulated nuclear factor erythroid 2–related factor 2 (NRF2) (Figure S2i). Concurrently, we observed a pronounced activation of the ER stress response, evidenced by the upregulation of key markers including protein kinase RNA‐like endoplasmic reticulum kinase (PERK), glucose‐regulated protein 78 (GRP78), activating transcription factor 6 (ATF6), and CCAAT/enhancer‐binding protein homologous protein (CHOP) (Figure S2f). This pathological cascade ultimately aggravated the inflammatory response, highlighted by the upregulation of interleukin‐1β (IL‐1β) and NLRP family pyrin domain containing 3 (NLRP3) (Figure S2i).

Escalating stress in MANF‐depleted LECs triggered an autophagic response, evidenced by increased RFP^+^GFP^−^ autolysosomes (Figure 3l,m, and Figure S2j). To characterize this process over time, we measured LC3‐II (reflecting autophagosome abundance) and p62 (an autophagic cargo) with or without Bafilomycin A1 (Baf A1) at 24, 48, and 72 h post‐knockdown. MANF knockdown elevated both basal and Baf A1‐induced LC3‐II levels, whereas p62 decreased at 24 h but progressively accumulated by 72 h (Figure 3n,o). These findings indicate that autophagic flux is initially enhanced but becomes overwhelmed under sustained stress. Consequently, LECs ultimately proceeded to apoptosis, which was corroborated by the upregulation of pro‐apoptotic proteins BCL‐2‐associated X protein (BAX) and cleaved caspase‐3 (Figure 3p,q) and by an increased ratio of propidium iodide (PI)‐positive to calcein‐AM‐positive cells (Figure 3r and Figure S2h).

Together, these findings delineate a pathogenic cascade in which MAM hyperassembly due to MANF depletion sets off aberrant Ca^2^^+^ flux, mitochondrial failure, and oxidative stress, leading to an overwhelmed autophagic response and culminating in LEC apoptosis.

### 2.4 Mitochondrial Defects Under Microinflammatory Stress Are Alleviated by MANF Restoration

Building on the pivotal role of MAM‐localized MANF in sustaining cellular homeostasis, we subsequently explored whether augmenting MANF expression could mitigate the microinflammatory stress‐induced pathogenic cascade in human LECs.

In addition to the aforementioned reversion of MAM hyperassembly, MANF overexpression in MCP‐1‐exposed SRA 01/04 LECs also effectively suppressed the progression toward apoptosis. Both mitochondrial Ca^2^^+^ uptake and ER Ca^2+^ release were restored to near‐normal levels (Figure 4a,b), accompanied by the recovery of protein expression for key Ca^2^^+^‐handling components—IP3R, VDAC, and MCU (Figure S2c,d). Mitochondrial function was significantly rescued, as evidenced by the recovery of ΔΨm (Figure 4c,d,f,g), restored cellular ATP levels (Figure 4e), and the reduction in mitochondrial superoxide (Figure 4h,j) and overall cellular ROS productions (Figure 4i and Figure S2e). The compromise of local redox homeostasis and the aggravated inflammatory response were also attenuated as shown by elevated NRF2, and reduced NOX and other inflammatory markers following MANF overexpression (Figure S2i and Figure 4o,p). Consistent with its canonical cytoprotective role, MANF depletion induced pronounced ER stress (evidenced by elevated GRP78/BiP, Cleaved ATF6, CHOP, and p‐eIF2α). Crucially, this severe ER stress burden was prominently suppressed by MANF overexpression in vitro (Figure S2g), underscoring the indispensable role of MANF in maintaining ER homeostasis in highly myopic cataracts.

**FIGURE 4:**
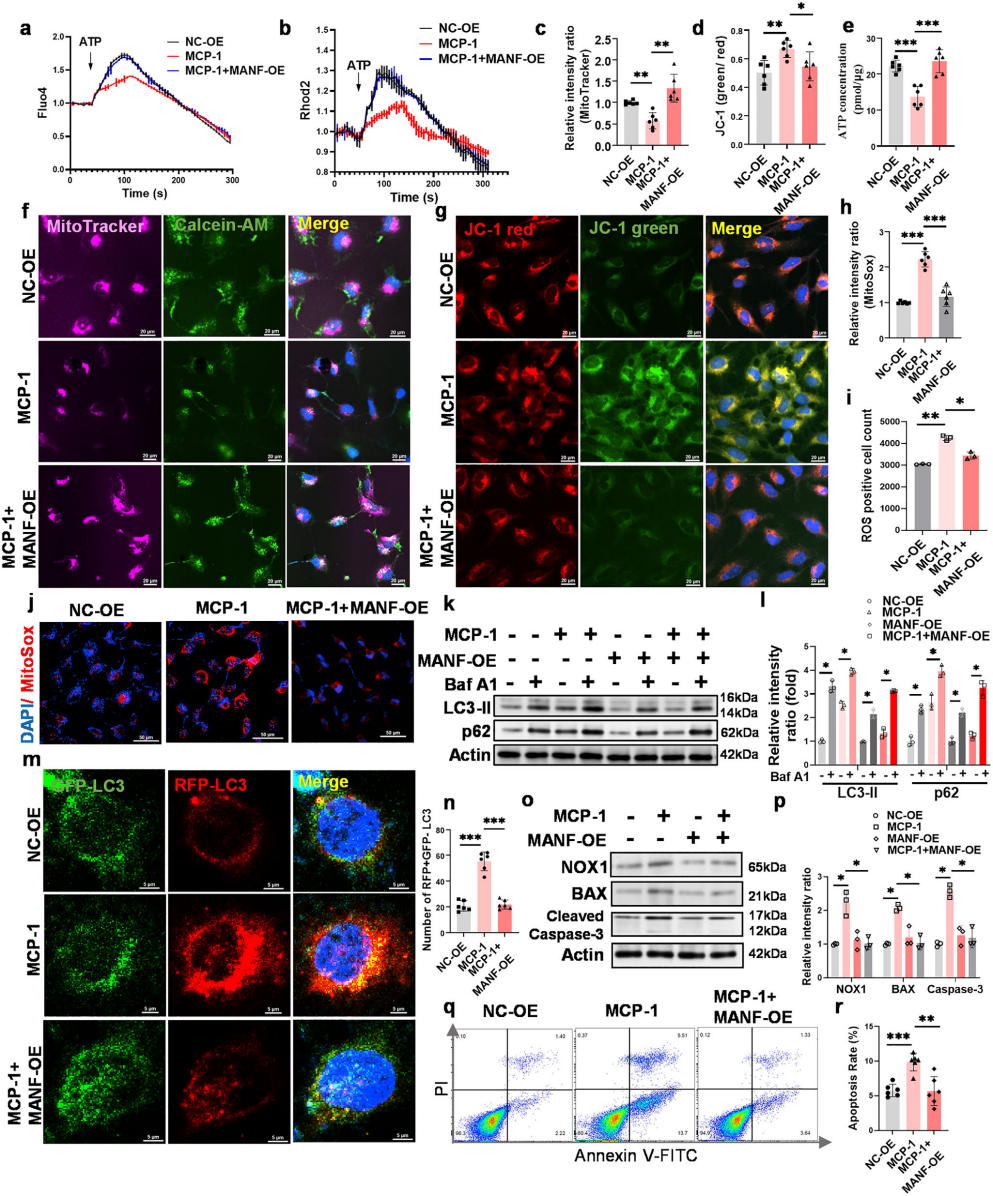
MANF protects LECs against mitochondrial defects and the ultimate apoptosis due to microinflammatory stress. (a,b) Effect of MANF restoration on endoplasmic reticulum (ER) Ca^2^^+^ release and mitochondrial Ca^2^^+^ uptake dynamics in SRA 01/04 human lens epithelial cells (LECs) exposed to MCP‐1 (100 nm, 48 h) to mimic the inflammatory microenvironment of highly myopic eyes, following ATP stimulation (*n* = 3). (c,d,f,g) Assessment of mitochondrial membrane potential (ΔΨm) by multiple methods in MCP‐1‐exposed LECs with or without MANF restoration: (c,f) MitoTracker (magenta) and Calcein‐AM (green) co‐staining (*n* = 6) and (d,g) JC‐1 staining (quantified by green/red fluorescence ratio, *n* = 6), Scale bars: 20 µm. (e) Cellular ATP content (*n* = 6). (h,j) Mitochondrial superoxide detected by MitoSOX staining (*n* = 6, scale bars: 50 µm). (i) Total cellular reactive oxygen species (ROS) measured by flow cytometry (*n* = 3). (k,l) LC3‐II and p62 protein levels analyzed by Western blot with or without 100 nm Bafilomycin (Baf) A1 (*n* = 3). (m,n) Analysis of autophagic flux in SRA 01/04 LECs expressing red fluorescent protein (RFP)‐green fluorescent protein (GFP)‐microtubule‐associated proteins 1A/1B light chain 3B (LC3). Autolysosomes (RFP + GFP‐LC3 puncta, red‐only) were quantified (*n* = 6). Scale bar: 5 µm. (o,p) Protein level changes of NADPH oxidase 1 (NOX1), BCL2‐associated X protein (BAX), and cleaved Caspase‐3 (*n* = 3) with MANF overexpression in MCP‐1‐treated LECs. (q,r) Apoptosis rate measured by flow cytometry using fluorescein isothiocyanate (FITC) Annexin V staining (*n* = 6). All data are presented as mean ± SD. Statistical significance was determined using two‐sided one‐way ANOVA with Tukey's multiple comparisons test (c,d,e,h,i,n,r) or two‐sided two‐way ANOVA with Šídák’ s multiple comparisons test (l,p). ^*^ *p* < 0.05, ^**^ *p* < 0.01, ^***^ *p* < 0.001.

Consistent with mitochondrial recovery, autophagic activity was markedly alleviated upon MANF restoration. In MCP‑1‑treated LECs, MANF overexpression reduced both basal and Baf A1‑induced LC3‑II levels, and simultaneously lowered p62 expression (Figure 4k,l), indicating normalization of autophagic flux and cargo clearance. This was further supported by a decreased formation of RFP^+^GFP^−^ autolysosomes (Figure 4m,n). Accordingly, expression of pro‑apoptotic proteins BAX and cleaved caspase‑3 was downregulated (Figure 4o,p), and the apoptotic cell ratio was reduced (Figure 4q,r).

Collectively, these findings demonstrate that restoring MANF rectifies the core pathological features driven by microinflammation, primarily through the recovery of mitochondrial‐ER Ca^2+^ transfer and mitochondrial bioenergetics.

### 2.5 MAM Remodeling and Disrupted Calcium Signaling via Ubiquitination‐Mediated Degradation of SERCA2 by MANF

To elucidate the molecular mechanism through which MANF regulates Ca^2^^+^ homeostasis, we performed co‐immunoprecipitation (Co‐IP) coupled with mass spectrometry in SRA 01/04 LECs. This process identified 213 proteins that specifically interact with MANF, 23 of which overlapped with the known MAM proteome. Bioinformatics analysis revealed SERCA2 as the top candidate, exhibiting the highest MANF‐binding score (Figure 5a), followed by AMP‐activated protein kinase (AMPK). The interaction between MANF and SERCA2 was bidirectionally validated by Co‐IP in lens epithelia from both HMC patients and HM mice (Figure 5b,c). GST pull‐down assay using purified proteins demonstrated a direct physical interaction between MANF and SERCA2 in vitro (Figure 5d). Immunofluorescence staining further confirmed their co‐localization in SRA 01/04 LECs (Figure 5e). SERCA2 is an ER‐resident membrane protein that plays a central role in cellular Ca^2^^+^ homeostasis. Based on this notion, we hypothesized that MANF modulates MAM‐associated Ca^2^^+^ flux through its interaction with SERCA2 in HMC. Several lines of evidence support this hypothesis. First, subcellular fractionation showed that microinflammatory stress triggered the enrichment of MANF and SERCA2 to MAM compartments (Figure S3h). Second, while SERCA2 mRNA levels showed no significant difference (Figure 5f), its protein level was significantly upregulated in HMC (Figure 5g–j). Third, MANF modulation did not alter SERCA2 mRNA (Figure 5k) but could bidirectionally regulate its protein abundance: MANF knockdown increased SERCA2 protein levels in LECs, whereas MANF overexpression downregulated them under both physiological and MCP‐1 treated conditions (Figure 5l,m and Figure S3c,d).

**FIGURE 5:**
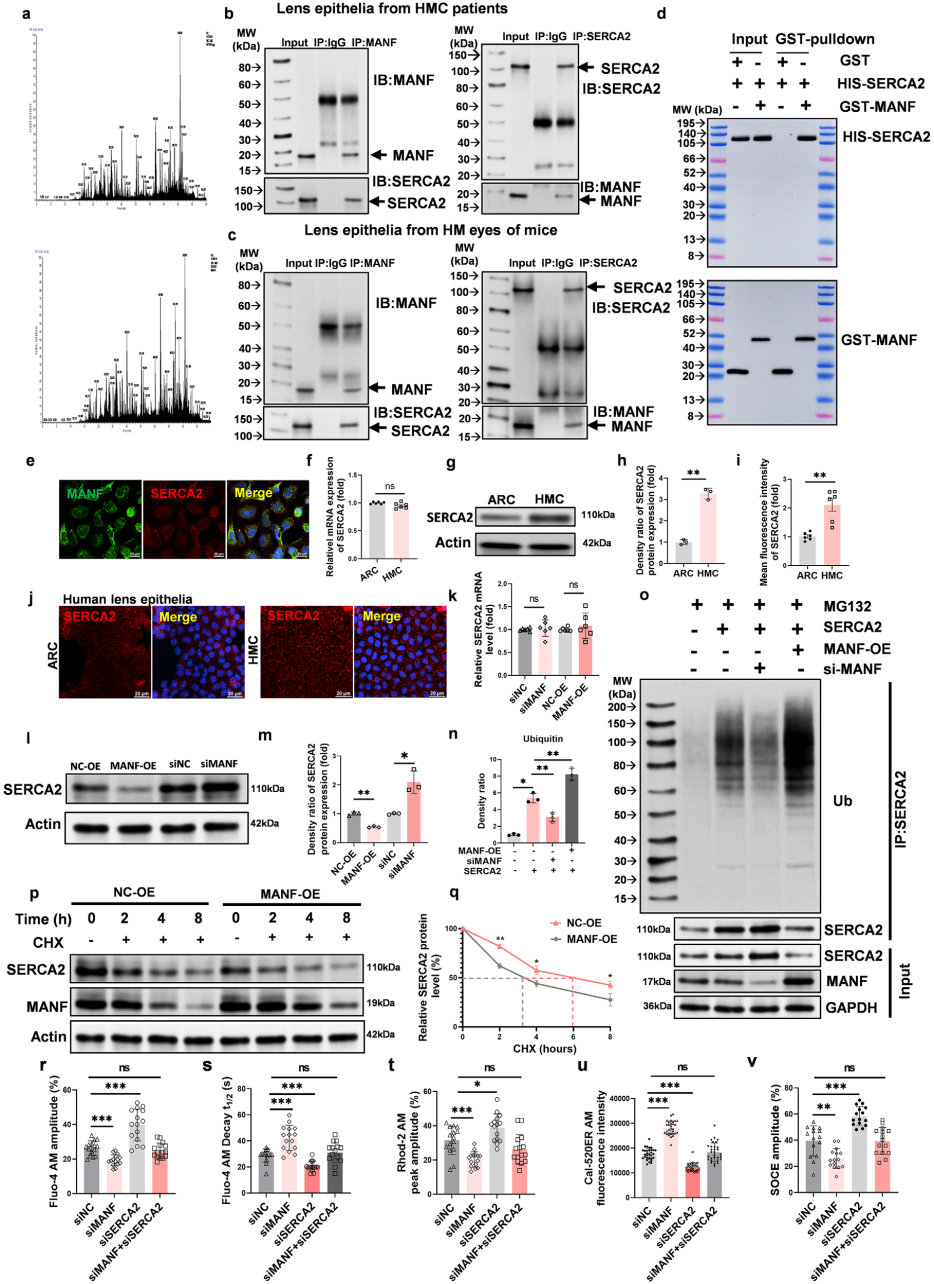
MANF modulates SERCA2 expression via ubiquitination‐mediated degradation. (a) Liquid chromatography with tandem mass spectrometry (LC‐MS/MS) analysis identified sarco/endoplasmic reticulum calcium ATPase 2 (SERCA2) as the top MANF‐interacting protein in SRA 01/04 human lens epithelial cells (LECs), followed by AMP‐activated protein kinase (AMPK). (b,c) Co‐immunoprecipitation (Co‐IP) bidirectionally validates the MANF‐SERCA2 interaction in lens epithelia from both highly myopic cataract (HMC) patients and HM eyes of mice. (d) A glutathione S‐transferase (GST) pull‐down assay revealed that MANF and SERCA2 interact in vitro. (e) Immunofluorescence (IF) staining showing co‐localization of MANF (green) and SERCA2 (red) in SRA 01/04 LECs. Scale bar: 20 µm. (f) SERCA2 mRNA level in lens epithelia from HMC and ARC patients (*n* = 6). (g,h,i,j) MANF protein expression in lens epithelia from HMC and ARC patients (*n* = 3 for western blot and *n* = 6 for IF). Scale bars: 20 µm. (k) SERCA2 mRNA level in SRA 01/04 LECs after modulation of MANF expression (*n* = 6). (l,m) Western blot and quantification of SERCA2 protein levels in LECs following MANF overexpression or knockdown (*n* = 3). (n,o) IP results showing increased SERCA2 ubiquitination and decreased SERCA2 protein levels in MANF‐overexpressing cells, whereas MANF knockdown reduces SERCA2 ubiquitination (*n* = 3). Ubiquitin signals were quantified by densitometry and normalized to the control group average. (p,q) Evaluation of SERCA2 protein stability over time by Western blot in MANF overexpressing LECs after treatment with the protein synthesis inhibitor cycloheximide (CHX, 100 µm) (*n* = 3). Protein decay rates were analyzed. (r—v) Multi‐dimensional Ca^2^^+^ dynamics in LECs transfected with siNC, siMANF, siSERCA2, or co‐transfected with siMANF and siSERCA2. Parameters include: (r) Cytosolic Ca^2^^+^ transient amplitude (Fluo‐4 AM) upon ATP stimulation, calculated as Δ*F*  = *F*_peak_  − *F*_baseline_ (*n*  =  15). (s) Cytosolic Ca^2^^+^ decay half‐life (t1/2) reflecting clearance rate (*n* = 15); (t) Mitochondrial Ca^2^^+^ uptake amplitude (Rhod‐2 AM), reflecting ER‐mitochondria Ca^2^^+^ transfer efficiency at MAMs and calculated as Δ *F*_mito_ = *F*_peak, mito_  − *F*_baseline, mito_ (*n*  =  15); (u) ER resting Ca^2^^+^ load measured by Cal‐520ER AM, expressed as baseline mean fluorescence intensity (*n*  =  30); and (v) Store‐operated Ca^2^^+^ entry (SOCE) amplitude, quantified after Thapsigargin (TG) treatment and Ca^2^^+^ re‐addition, using $\frac{F_{\mathrm{peak}}-F_{\mathrm{TG}\;\mathrm{bottom}}}{F_{0}}$ (*n*  =  15). Data are presented as mean ± SD. Statistical significance was determined using an unpaired t‐test (f,h,i), two‐sided one‐way ANOVA with Tukey's multiple comparisons test (k,m,n,r–v) or two‐sided two‐way ANOVA with Šídák’ s multiple comparisons test (q). n represents the number of biological replicates. ns = not significant, ^*^ *p* < 0.05, ^**^ *p* < 0.01, ^***^ *p* < 0.001.

To define the structural basis of this interaction, we performed high‐resolution protein‐protein molecular docking using the HDOCK algorithm. The computational modeling yielded a stable complex (docking score: –251.87), revealing that intraluminal MANF perfectly docks onto the luminal‐facing domains of SERCA2b. This interaction is stabilized by an intricate network of 9 hydrogen bonds (e.g., SERCA2 HIS284 with MANF GLN100; GLY277 with ARG15) and a critical salt bridge (SERCA2b HIS278 and MANF GLU47) (Figure S3b). This structural modeling strongly supports that intraluminal MANF directly physically engages the luminal architecture of SERCA2. Subcellular fractionation also revealed that MANF and SERCA2, along with AMPK and a key structural protein that builds MAM‐ mitofusin‐2 (MFN2), localize to MAMs and further enriche under inflammatory stress, suggesting a stress‐induced translocation from the cytosol to MAM contact sites (Figure S3h). Co‐IP experiments confirmed that SERCA2 forms a complex with both AMPK and MFN2 at MAMs (Figure S3j,k), supporting its role as a core component of a MAM‐localized regulatory complex that includes MANF.

Given our finding that MANF engages the luminal loops of SERCA2, its regulation of SERCA2 degradation implies the involvement of the canonical Endoplasmic Reticulum‐Associated Degradation (ERAD) pathway, wherein luminal binding triggers cytosolic ubiquitination. To test this post‐translational regulation, we performed in vitro ubiquitination assays. The immunoprecipitation results demonstrated that MANF bidirectionally dictates the polyubiquitination status of SERCA2: MANF overexpression significantly increased SERCA2 ubiquitination and consequently decreased its total protein level, whereas MANF knockdown markedly reduced SERCA2 ubiquitination, leading to its pathological accumulation (Figure 5n,o). Cycloheximide chase assays further corroborated these findings, showing that MANF overexpression significantly shortened the half‐life of the SERCA2 protein (Figure 5p,q).

To functionally validate that this MANF‐SERCA2 regulatory axis dictates global Ca^2^^+^ disturbances, we performed a comprehensive epistasis analysis using a double‐knockdown strategy. We evaluated the specific Ca^2^^+^ signatures across four groups: siNC, siMANF, siSERCA2, and double knockdown (siMANF + siSERCA2) (Figure 5r–v and Figure S3e–g). Knockdown of MANF, which impairs SERCA2 degradation leading to its hyperactivity, resulted in marked ER Ca^2^^+^ overload (Figure 5u). This was accompanied by a blunted cytosolic Ca^2^^+^ transient amplitude (Fluo‐4 AM) with a prolonged decay half‐life (Figure 5r,s), decreased mitochondrial Ca^2^^+^ uptake (Rhod‐2 AM) (Figure 5t), and suppressed Store‐Operated Calcium Entry (SOCE) (Figure 5v). Conversely, silencing SERCA2 alone produced the exact opposite phenotypic spectrum: depleted ER Ca^2^^+^ stores, amplified cytosolic and mitochondrial Ca^2^^+^ transients, accelerated cytosolic Ca^2^^+^ clearance, and enhanced SOCE. Strikingly, simultaneous silencing of SERCA2 and MANF completely neutralized the aberrant Ca^2^^+^ signaling induced by MANF deficiency, rescuing the ER, cytosolic, and mitochondrial Ca^2^^+^ fluxes, as well as SOCE, back to near‐baseline levels.

Collectively, these data indicate that under microinflammatory stress in HM eyes, downregulation of MANF impairs ubiquitination‐mediated degradation of SERCA2, leading to its pathological accumulation at MAMs, which may underlie the disruption of physiological Ca^2+^ homeostasis in LECs.

### 2.6 Lens‐Specific MANF Deletion in Vivo Recapitulates the Pathogenic Cascade of HMC in Mice

To establish the in vivo relevance of our findings, we generated lens‐specific *Manf* knockdown mice (*MANF* ^flox/flox^; Prox1‐cre‐ERT2, termed MANF cKD) (Figure 6a). Tamoxifen induction efficiently ablated MANF expression in the lens, which was confirmed at both mRNA and protein levels. Concurrently, a significant aberrant upregulation of SERCA2 was also observed (Figure 6b–f,h,i).

**FIGURE 6:**
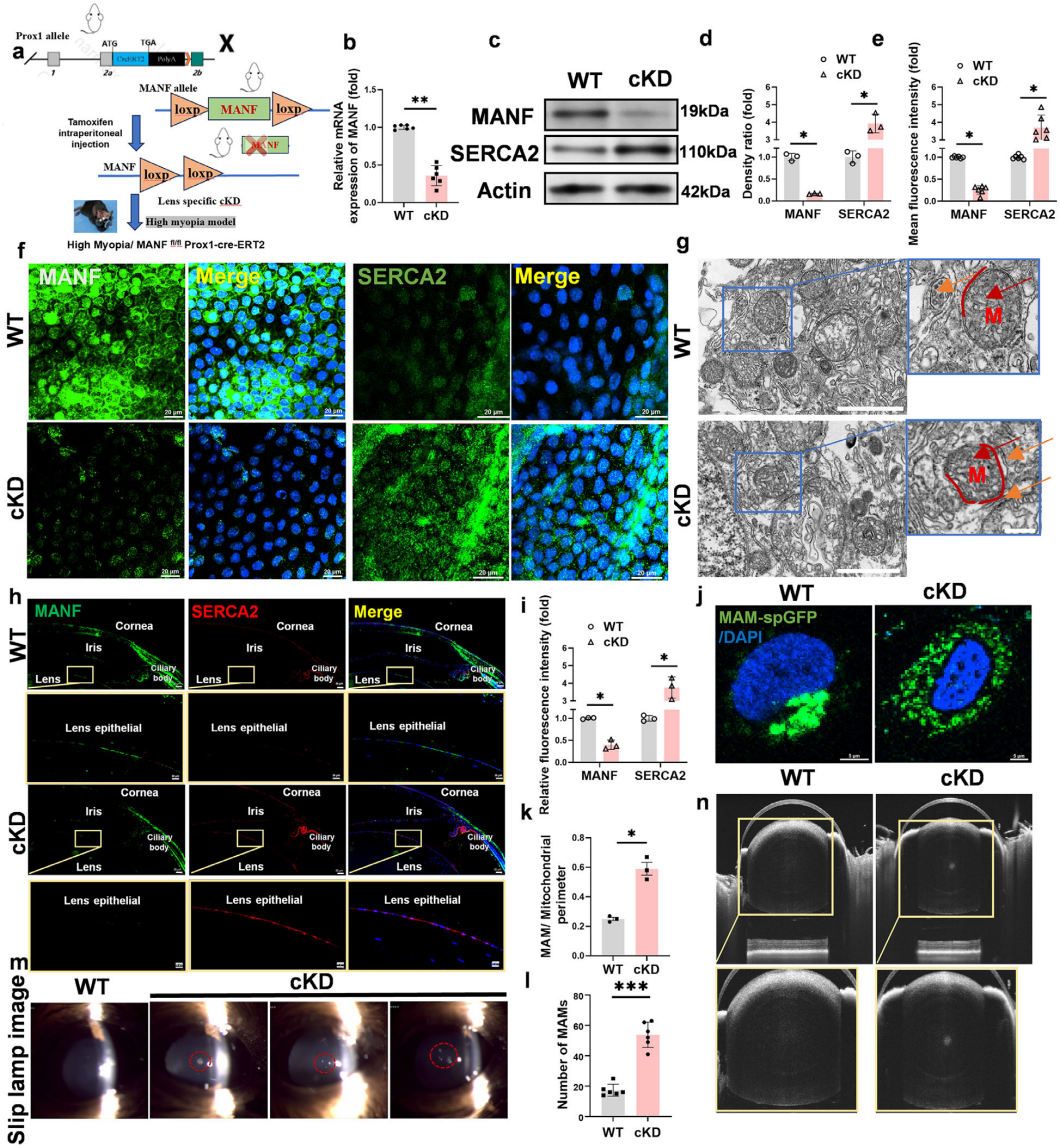
Lens‐specific MANF depletion accelerates cataractogenesis in mice. (a) Schematic of generating lens‐specific MANF conditional knockdown (cKD) mice generated via Cre‐LoxP system. Mice with genotype Prox1‐CreERT^ct2/wt^, MANF^flox/flox^ (cKD) were treated with tamoxifen at 4 weeks to induce Cre recombinase expression and excise the MANF gene. MANF^flox/flox^ mice served as wild‐type (WT) controls. Subsequently, high myopia (HM) was induced in the right eye of mice using a −10 diopter (D) defocus lens at five weeks. (b) mRNA levels of MANF in lens epithelia from the indicated groups (WT and cKD mice) measured by qRT‐PCR (*n* = 6). (c–f) Protein expression of MANF and SERCA2 in lens epithelia from each group (Western blot *n* = 3, immunofluorescence staining, IF *n* = 6). Nuclei were counterstained with DAPI. Scale bars: 20 µm. (h,i) IF co‐staining of MANF (green) and SERCA2 (red) in lens tissue sections. Nuclei were stained with DAPI (blue). The yellow boxes indicate the regions shown in the magnified views below. Scale bars: 50 µm (overview), 20 µm (magnified views) (*n* = 3). (g,k) Representative transmission electron microscopy (TEM) images of mitochondria‐associated membranes (MAMs) in lens epithelia, with quantification of MAM length (*n* = 3). Red lines delineate the closely apposed regions between mitochondria (M; indicated by red arrows) and the endoplasmic reticulum (ER; indicated by orange arrows). Scale bars: 1 µm (overview), 100 nm (enlargements). (j,l) IF analysis of MAMs in primary lens epithelial cells isolated from the indicated groups. Cells were transfected with a split‐green fluorescent protein (spGFP) reporter to label MAMs (green); nuclei were stained with DAPI (blue) (*n* = 6). Scale bars: 5 µm. (m) Representative slit‐lamp images of mouse lenses from the WT and cKD groups. The red circles highlight regions of lens opacity (cataract). (n) Representative swept‐source optical coherence tomography (OCT) scans of mouse lenses from the WT and cKD groups. Yellow boxes indicate the regions shown in the enlarged views. Data are presented as mean ± SD. Statistical significance was determined using an unpaired t‐test (b,k,l), or Mann‐Whitney U test (d,e,i). n represents biological replicates. ^*^ *p* < 0.05, ^**^ *p* < 0.01, ^***^ *p* < 0.001.

Compared to control littermates (*MANF* ^flox/flox^; *cre^−^*), primary LECs from 8‐week‐old MANF cKD mice exhibited pronounced MAM hyperassembly. This was evidenced by increased MAM length and enhanced fluorescence intensity of the MAM‐specific spGFP reporter (Figure 6g,j–l). These mice also developed progressive lens opacification, characterized by a significant increase in lens nucleus density, which was also observed in slit‐lamp photographs and anterior segment optical coherence tomography (AS‐OCT) images (Figure 6m,n).

We further characterized the functional consequences of MAM disruption in this model. Primary LECs isolated from MANF cKD mice showed severely impaired inter‐organelle Ca^2^^+^ transfer. Upon ATP stimulation, both cytosolic and mitochondrial Ca^2^^+^ peaks were markedly attenuated (Figure 7a,b) concomitant with downregulation of key ER‐mitochondria Ca^2^^+^ handling proteins (IP3R, VDAC, and MCU) (Figure S4a,b).

**FIGURE 7:**
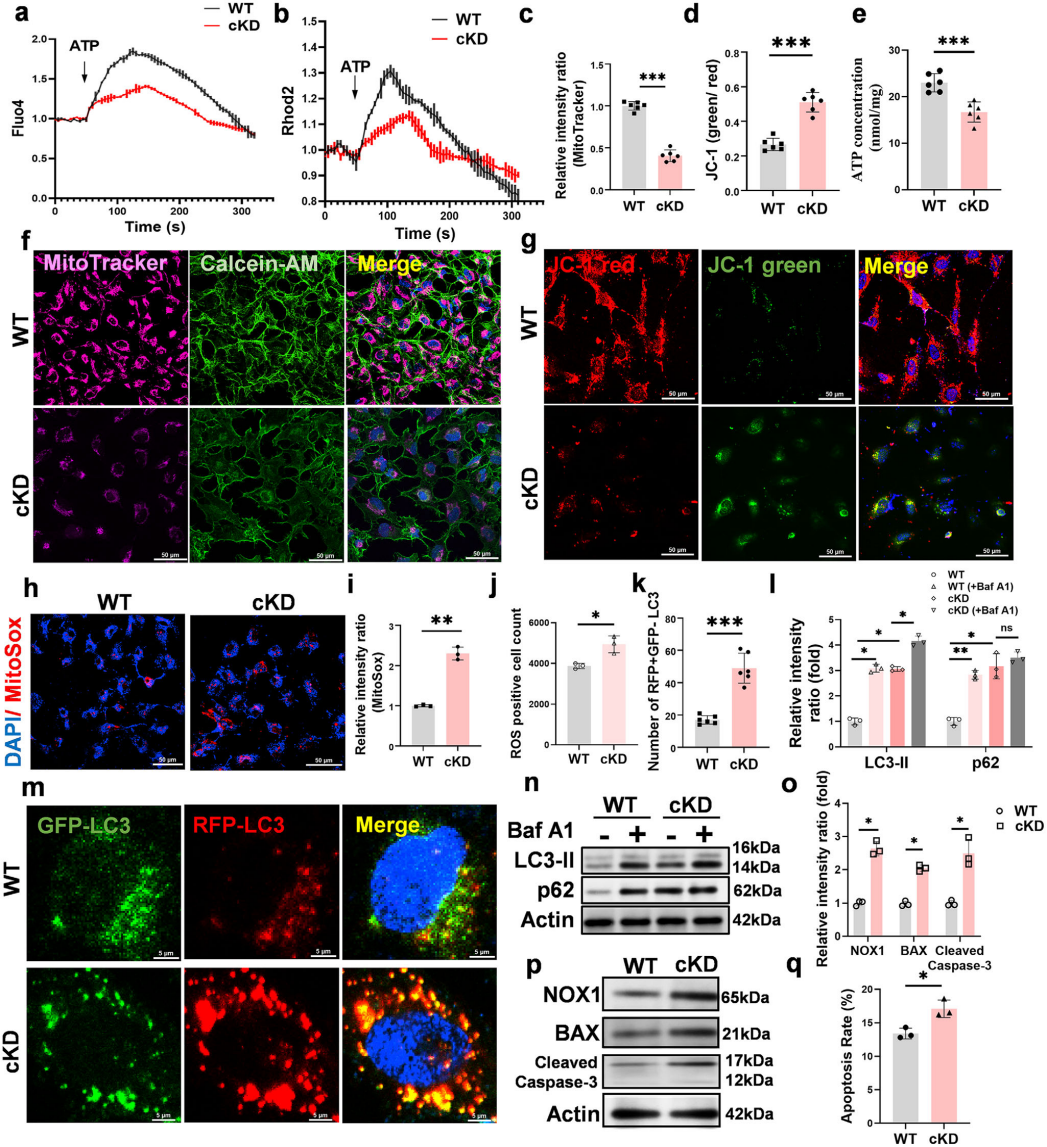
Lens‐specific MANF depletion Induces Mitochondrial Dysfunction and Apoptosis in Primary Lens Epithelial Cells of mice. Primary lens epithelial cells isolated from WT and MANF cKD mice were subjected to the following assays: (a,b) Representative curves of fluorescence density over time reflecting cytosolic (Fluo‐4 AM) and mitochondrial (Rhod‐2 AM) Ca^2^^+^ levels after ATP stimulation (*n* = 3). (c,d,f,g) Mitochondrial membrane potential (ΔΨm) assessed by (c,f) MitoTracker and Calcein‐AM co‐staining (*n* = 6) and (d,g) JC‐1 staining (green/red ratio, *n* = 6), Scale bars: 50 µm. (e) Cellular ATP content (n = 6). (h–j) Assessment of oxidative stress: (h,i) mitochondrial superoxide production detected by MitoSOX Red staining (*n* = 3, scale bars: 50 µm) and (j) total cellular reactive oxygen species (ROS) levels measured by flow cytometry (*n* = 3). (k,m) Analysis of autophagic flux using RFP+GFP‐LC3 Autolysosomes (RFP^+^GFP^−^ puncta) were quantified (*n* = 6). Scale bar: 5 µm. (l,n) Western blot analysis of LC3‐II and p62 levels with/without 100 nM Bafilomycin (Baf) A1 (*n* = 3). (o,p) Protein expression of apoptosis‐related markers NADPH oxidase 1 (NOX1), BCL2‐associated X protein (BAX), and cleaved Caspase‐3 (*n* = 3). (q) Apoptosis rate measured by flow cytometry (FITC Annexin V staining, *n* = 3). All data are presented as mean ± standard deviation (SD). Statistical significance was determined using an unpaired t‐test (c,d,e,k,q), Mann‐Whitney U test (i,j), two‐sided one‐way ANOVA with Tukey's multiple comparisons test (l) or two‐sided two‐way ANOVA with Šídák’ s multiple comparisons test (o); n represents the number of biological replicates. ^*^ *p* < 0.05, ^**^ *p* < 0.01, ^***^ *p* < 0.001.

Subsequent to this Ca^2^^+^ dysregulation, LECs from MANF cKD mice replicated the cascade of cellular stress and dysfunction identified in HMC. Pronounced mitochondrial membrane potential impairment (Figure 7c,d,f,g and Figure S4e,f), decrease in cellular ATP levels (Figure 7g), excessive ROS production (Figure 7h–j and Figure S4i), ER stress (Figure S4k), elevated autophagy (Figure 7k–n), cell cycle arrest (Figure S4c,d), and ultimately, apoptosis (Figure S4j and Figure 7o–q) were seen. In addition, lens epithelia of MANF cKD mice also displayed changes in SERCA2‐interacting proteins including MFN2 (Figure S4g,h).

Collectively, these in vivo data validate the pathogenic cascade linking MANF deficiency to MAM remodeling, mitochondrial dysfunction, and subsequent cellular demise, thereby underpinning the pathogenesis of HMC.

### 2.7 In Vivo AAV2‐Mediated MANF Restoration Ameliorates HMC Formation in Mice

To evaluate the therapeutic potential of MANF, we delivered an AAV2 vector expressing MANF (AAV2‐MANF) or an empty vector control (AAV2‐EV) via intravitreal injection to lens‐specific MANF cKD mice subjected to HM modeling (Figure 8a). This treatment successfully restored MANF expression and reversed the pathological accumulation of SERCA2 in the lens (Figure 8b,c). Notably, the nuclear cataracts observed in HM MANF cKD mice, which persisted in the AAV2‐EV treated eyes, were significantly alleviated following MANF restoration (Figure 8t).

**FIGURE 8:**
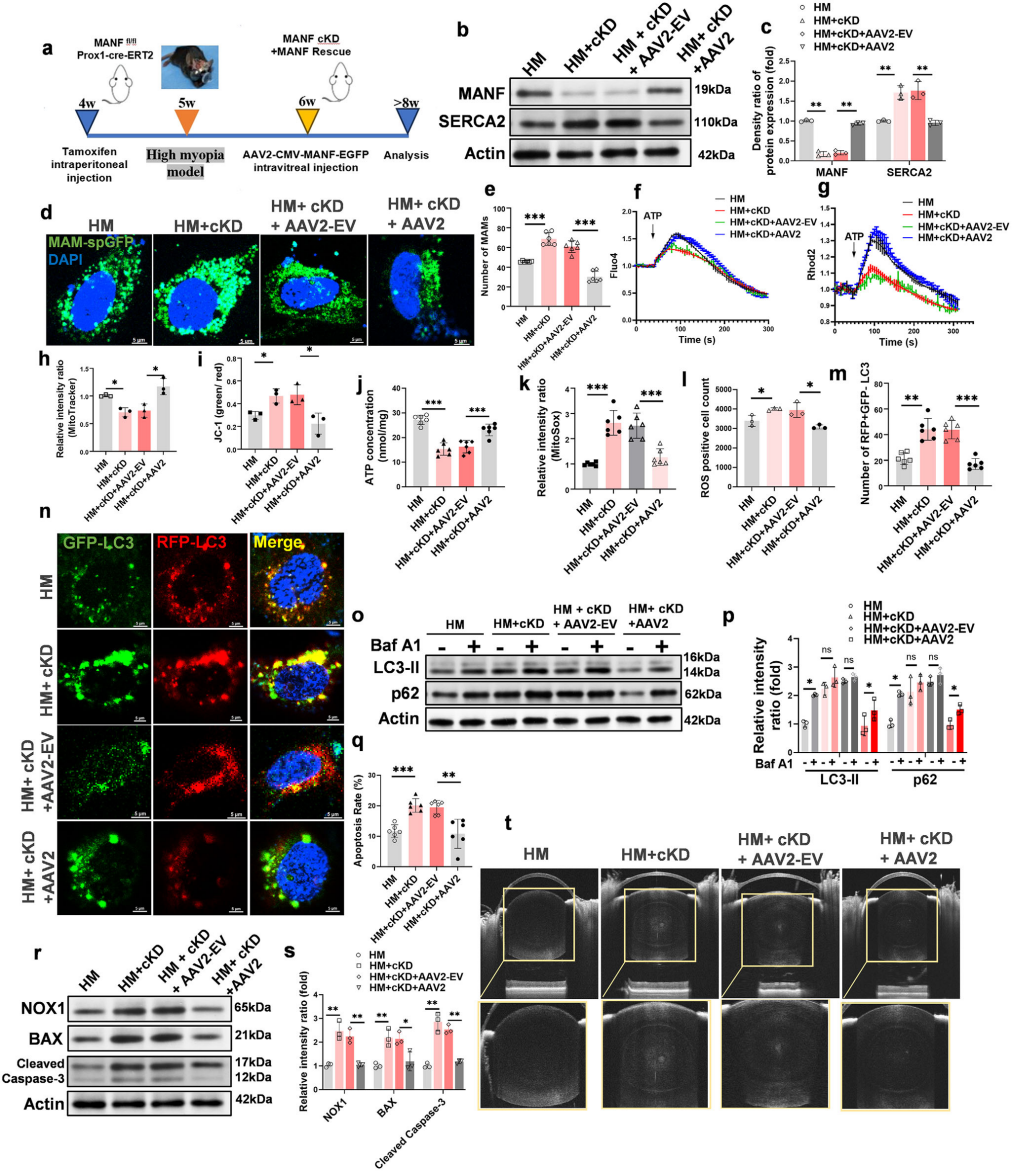
MANF restoration ameliorates cataract formation in HM mice. (a) Schematic timeline of the experimental procedure. In Prox1‐CreERT^ct2/wt^, MANF^flox/flox^ (cKD) mice, lens‐specific MANF deletion was first induced by intraperitoneal injection of tamoxifen. Subsequently, high myopia (HM) was induced in the right eye using a −10 diopter (D) defocus lens, followed by an intravitreal injection of an adeno‐associated virus serotype 2 (AAV2) vector expressing MANF and enhanced green fluorescent protein (EGFP) under the cytomegalovirus (CMV) promoter (AAV2‐CMV‐MANF‐EGFP) to achieve MANF overexpression, or an empty vector control (AAV2‐EV). (b,c) MANF and SERCA2 protein levels in lens epithelia from the indicated groups (*n* = 3). (d–s) Primary lens epithelial cells (LECs) isolated from HM, HM + cKD, HM+cKD+AAV2‐EV, and HM+cKD+AAV2 mice group were analyzed as follows: (d,e) Immunofluorescence (IF) imaging of mitochondria‐associated membranes (MAMs). Cells were transfected with a split‐green fluorescent protein (spGFP) MAM reporter (green); nuclei were counterstained with DAPI (blue) (*n* = 6). Scale bars: 5 µm. (f,g) Representative curves of fluorescence density over time reflecting cytosolic (Fluo‐4 AM) and mitochondrial (Rhod‐2 AM) Ca^2^^+^ levels after ATP stimulation (*n* = 3). (h), i Mitochondrial membrane potential (ΔΨm) assessed by MitoTracker and Calcein‐AM co‐staining (*n* = 6) and JC‐1 staining (green/red ratio, *n* = 6). (j) Cellular ATP content (*n* = 6). (k) Mitochondrial superoxide production detected by MitoSOX staining (*n* = 6). (l) Total cellular ROS levels measured by flow cytometry (*n* = 3). (m,n) Analysis of autophagic flux using RFP+GFP‐LC3 autolysosomes, and puncta were quantified (*n* = 6). Scale bar: 5 µm. (o,p) Western blot analysis of LC3‐II and p62 levels with/without 100 nm Bafilomycin (Baf) A1 (*n* = 3). (q) Apoptosis rate measured by flow cytometry (FITC Annexin V staining, *n* = 6). (r,s) Protein expression of apoptosis‐related markers NADPH oxidase 1 (NOX1), BCL2‐associated X protein (BAX), and cleaved Caspase‐3 (*n* = 3). (t) Representative swept‐source optical coherence tomography (OCT) scans of lenses from HM, HM + MANF cKD‐EV, and HM + MANF cKD + AAV2 ‐MANF rescue groups. Yellow boxes indicate the regions shown in the enlarged views. All data are presented as mean ± SD. Statistical significance was determined using two‐sided one‐way ANOVA with Tukey's multiple comparisons test (e,h–m,q) or two‐sided two‐way ANOVA with Šídák’ s multiple comparisons test (c,p,s). n represents biological replicates. ^*^ *p* < 0.05, ^**^ *p* < 0.01, ^***^ *p* < 0.001.

Mechanistically, compared to the AAV2‐EV controls, in vivo MANF restoration effectively reversed the pathogenic cascade previously identified in HMC patients and MANF cKD mice. It normalized the aberrant MAM structure by reducing abnormal increased MAM contacts (Figure 8d,e). Concurrently, Ca^2+^ homeostasis was fully rescued, as evidenced by restored expression of key Ca^2^^+^‐handling proteins (IP3R, MCU, VDAC) (Figure S5a,b) and the recovery of diminished cytosolic and mitochondrial Ca^2^^+^ peaks (Figure 8f,g). Further analysis demonstrated that MANF restoration also recovered ΔΨm (Figure S5h,i and Figure 8h,i), boosted cellular ATP production (Figure 8j), attenuated oxidative stress (Figure S5d,f,g and Figure 8k,l), mitigated autophagy (Figure 8m–p) and returned apoptosis rates to near‐normal levels (Figure 8q–s and Figure S5e).

Collectively, these results demonstrate that AAV2‐mediated MANF rescue therapy can rectify the structural and functional impairment of MAMs, halt the downstream pathogenic cascade, and ultimately prevent the nuclear opacifications in experimental HMC.

## 3 Discussion

In this study, we bridge the fundamental mechanistic gap between extracellular microinflammatory stress and intracellular organelle failure, utilizing HMC as a robust biological paradigm. Our work uncovers a previously unappreciated, non‐canonical defense mechanism orchestrated by MANF at the ER‐mitochondria interface. Expanding beyond its conventional identity as a generalized ER‐resident chaperone, we identify MANF as a critical luminal quality‐control sensor structurally localized at MAMs. In response to MCP‐1‐driven microinflammation, MANF physically engages the trans‐membrane pump SERCA2, promoting its ubiquitin‐mediated degradation to dynamically safeguard ER‐to‐mitochondria Ca^2^^+^ homeostasis. We provide compelling evidence across human clinical specimens, cellular assays, and novel in vivo genetic models (*Manf* conditional knockdown and defocus‐induced high myopia mice) that microinflammation‐induced MANF deficiency triggers a devastating cascade. This cascade is characterized by pathological SERCA2 accumulation, detrimental MAM hyperassembly, severe Ca^2^^+^ flux dysregulation, overwhelmed autophagic clearance, and terminal mitochondrial collapse. Collectively, these findings shift the focus of microinflammation‐driven pathology from isolated organelle dysfunction to the profound disruption of inter‐organelle crosstalk, establishing the MANF‐SERCA2 interplay as a universal framework with broad therapeutic implications for microinflammation‐associated degenerative disorders beyond Ophthalmology.

Our work positions MANF as a critical molecular counterforce to the chronic microinflammatory stress that characterizes HM. Accumulating clinical and experimental evidence supports that the HM eye exists in a state of sustained, low‐grade inflammation, marked by cytokines such as MCP‐1 [19, 20, 21, 22]. This microenvironment imposes a persistent proteostatic and metabolic challenge on long‐lived cells like LECs. While microinflammation is a known initiator of ER stress and oxidative damage, its specific link to organelle communication failure in the lens has been elusive. Here, we identify the depletion of MANF as a pivotal event that transduces this inflammatory signal into profound cellular dysfunction.

Our findings, for the first time, fundamentally extend the understanding of MANF's cytoprotective role by pinpointing its critical function at the MAM interface, a strategic location for mitigating stress originating from both organelles. While MANF is recognized as an ER‐resident protein alleviating ER stress, its significant enrichment at MAMs [7, 46, 47], as revealed by our split‐GFP and subcellular fractionation assays, suggests a dedicated role in regulating organelle coupling. The pathological “hyperassembly” of MAMs upon MANF depletion is particularly instructive. Contrary to the notion that increased organelle contacts are inherently beneficial, our data align with emerging evidence across various diseases such as Alzheimer's disease and nonalcoholic steatohepatitis, where excessive, unregulated ER‐mitochondria tethering can disrupt precise Ca^2^^+^ signaling, promote cell death and disease deterioration [6, 7, 8, 48]. To distinguish whether this hyperassembly acts as a pathological driver or a secondary compensatory adaptation, we artificially tightened ER‐mitochondria contacts using MFN2 overexpression [49, 50]. This forced tethering autonomously phenocopied the mitochondrial bioenergetic collapse and apoptotic fate seen in MANF deficiency, demonstrating that excessive MAM contacts are inherently detrimental. While future studies utilizing purely inert synthetic linkers will be invaluable to completely isolate the spatial tethering variable, our current data firmly position MANF as an active stabilizer preventing pathogenic hyper‐tethering. This role provides a coherent mechanistic explanation for MANF's previously reported, yet unexplained, involvement in both ER and mitochondrial homeostasis.

At the molecular level, we unveiled the interaction between MANF and SERCA2 as a key regulatory node. Crucially, our biochemical and computational structural analyses resolve a long‐standing topological paradox regarding this interaction. We define MANF as a strictly ER‐intraluminal protein that physically docks onto the luminal‐facing loops of the transmembrane SERCA2 pump. This luminal engagement surprisingly facilitates the cytosolic polyubiquitination of SERCA2, a process that perfectly aligns with the canonical Endoplasmic Reticulum‐Associated Degradation (ERAD) pathway [51, 52]. In this context, we propose that intraluminal MANF serves as a highly sensitive conformational sensor. Upon microinflammatory stress, its binding to the luminal domains of hyperactive or stressed SERCA2 recruits transmembrane ERAD E3 ubiquitin ligases, which in turn catalyze the polyubiquitination of SERCA2 on the cytosolic side for targeted proteasomal clearance [53].

The observation that MANF promotes SERCA2 polyubiquitination and degradation represents a significant shift in paradigm. SERCA2, a crucial enzyme that actively pumps Ca^2+^ from the cell's cytoplasm back into the sarcoplasmic/endoplasmic reticulum (SR/ER), controls Ca^2+^ signals vital for muscle contraction/relaxation and cell function [41, 42, 43, 44, 54]. Its activity is traditionally regulated by post‐translational modifications and allosteric binding partners [54, 55, 56, 57]. Our discovery that its protein abundance is directly controlled by MANF via the ubiquitin‐proteasome system introduces a novel layer of regulation for cellular Ca^2^^+^ handling. Similarly in pulmonary hypertension, the pathogenesis involves the ubiquitin‐mediated degradation of SERCA2 via tribbles homolog 2, which induces ER stress and accelerates cell cycle, promoting pulmonary artery smooth muscle cell hyperproliferation, and inhibiting apoptosis [58]. Here under microinflammatory stress in HM eyes, reduced MANF leads to aberrant SERCA2 accumulation. Crucially, our epistatic rescue experiments unequivocally establish this SERCA2 accumulation as the primary causal effector of the global Ca^2^^+^ chaos, rather than a mere correlative event. MANF deficiency‐induced SERCA2 hyperactivity precipitates a severe ER Ca^2^^+^ overload [59]. According to established principles of Ca^2^^+^ signaling, such chronic ER overload subsequently desensitizes IP3R‐mediated Ca^2^^+^ release and acts upstream of secondary alterations in MAM‐associated channels (such as VDAC and MCU) [60, 61]. Consequently, this pathological cascade severely blunts ER‐to‐mitochondria Ca^2^^+^ transfer and suppresses store‐operated Ca^2^^+^ entry (SOCE). Strikingly, targeted depletion of SERCA2 in MANF‐deficient cells completely neutralizes these multi‐dimensional Ca^2^^+^ defects, restoring ER load, cytosolic transients, and mitochondrial uptake to physiological baselines. This mechanism elegantly connects the upstream proinflammatory signal (MCP‐1) to the downstream bioenergetic crisis (mitochondrial failure) through a defined protein‐protein interaction and degradation pathway.

The consequences of this MANF‐SERCA2 axis disruption integrate several established pillars of cataractogenesis. The ensuing mitochondrial depolarization, ATP depletion, and surge in ROS directly fuel oxidative damage, a long‐acknowledged driver of lens opacity [62, 63, 64, 65]. Simultaneously, impaired Ca^2^^+^ flux and energetic stress activate pro‐apoptotic pathways, committing LECs to apoptosis—a final common pathway in cataract formation. Notably, we observed an initial upregulation of autophagy in MANF‐depleted cells, likely a compensatory response to clear damaged organelles. By dynamically tracking p62 cargo clearance, we unveiled a biphasic autophagic response, beginning with an initial hyperactive, structurally intact clearance phase. However, under persistent metabolic stress, this protective mechanism is eventually overwhelmed by the sheer magnitude of unremitting MAM Ca^2^^+^ overload, ultimately committing cells to apoptotic death. Furthermore, the activation of the NLRP3 inflammasome in this context creates a vicious cycle, where mitochondrial dysfunction (e.g., mitochondrial ROS, cytochrome c release) fuels inflammation, which in turn further aggravates cellular stress. MANF restoration breaks this cycle by rectifying the initial mitochondrial defect, underscoring its role as a master regulator of integrated stress response in LECs.

The in vivo relevance of this pathway is firmly established by our lens‐specific MANF KD model. The MANF cKD mice phenocopy core features of human HMC, developing nuclear opacities.Furthermore, the recapitulation of the full pathogenic cascade—from MANF downregulation and MAM remodeling to mitochondrial failure and apoptosis—in primary LECs from these mice provides compelling causal evidence. Most importantly, the therapeutic potential is convincingly demonstrated by AAV‐mediated MANF gene delivery, which successfully preserved lens transparency, normalized MAM structure, and rescued mitochondrial bioenergetics in MANF cKD mice under myopic stress.

While this study delineates a coherent pathway from microinflammation to cataract, several questions remain. First, the precise mechanism by which MCP‐1 signaling suppresses MANF expression warrants further investigation, potentially involving specific transcription factors or epigenetic modifications. Second, while our study provides strong computational and biochemical evidence for MANF's luminal docking and ERAD‐mediated ubiquitination of SERCA2, identifying the specific transmembrane E3 ubiquitin ligase bridging this complex remains a vital area for future investigation. Third, our work primarily focuses on MCP‐1; other components of the HM microinflammatory soup may also converge on MANF regulation.

Our study establishes the MANF‐SERCA2 axis at the MAM interface as a pivotal transducer of extracellular microinflammatory stress into intracellular organelle dysfunction. Utilizing highly myopic cataract as a robust biological paradigm, we demonstrate that microinflammation‐induced MANF deficiency drives pathological MAM hyperassembly and terminal mitochondrial collapse. Strikingly, this deleterious cascade is entirely reversible in vivo via AAV2‐mediated targeted MANF gene delivery, which successfully normalizes MAM architecture, rescues mitochondrial function, and prevents cataractogenesis (Figure 9). In summary, this study establishes the MANF‐SERCA2 axis at the MAM interface as a critical pathway linking microinflammation to organelle dysfunction in HMC. By elucidating how microinflammatory stress disrupts inter‐organelle crosstalk, our findings not only enhance the understanding of cataractogenesis but also propose the MANF‐SERCA2 interaction as a promising therapeutic target for mitigating microinflammation‐driven tissue degeneration across various diseases. This research underscores the potential for pharmacological strategies aimed at restoring MANF function to counteract the deleterious effects of chronic inflammation, thereby offering new avenues for treatment in both ophthalmology and broader metabolic disorders.

**FIGURE 9:**
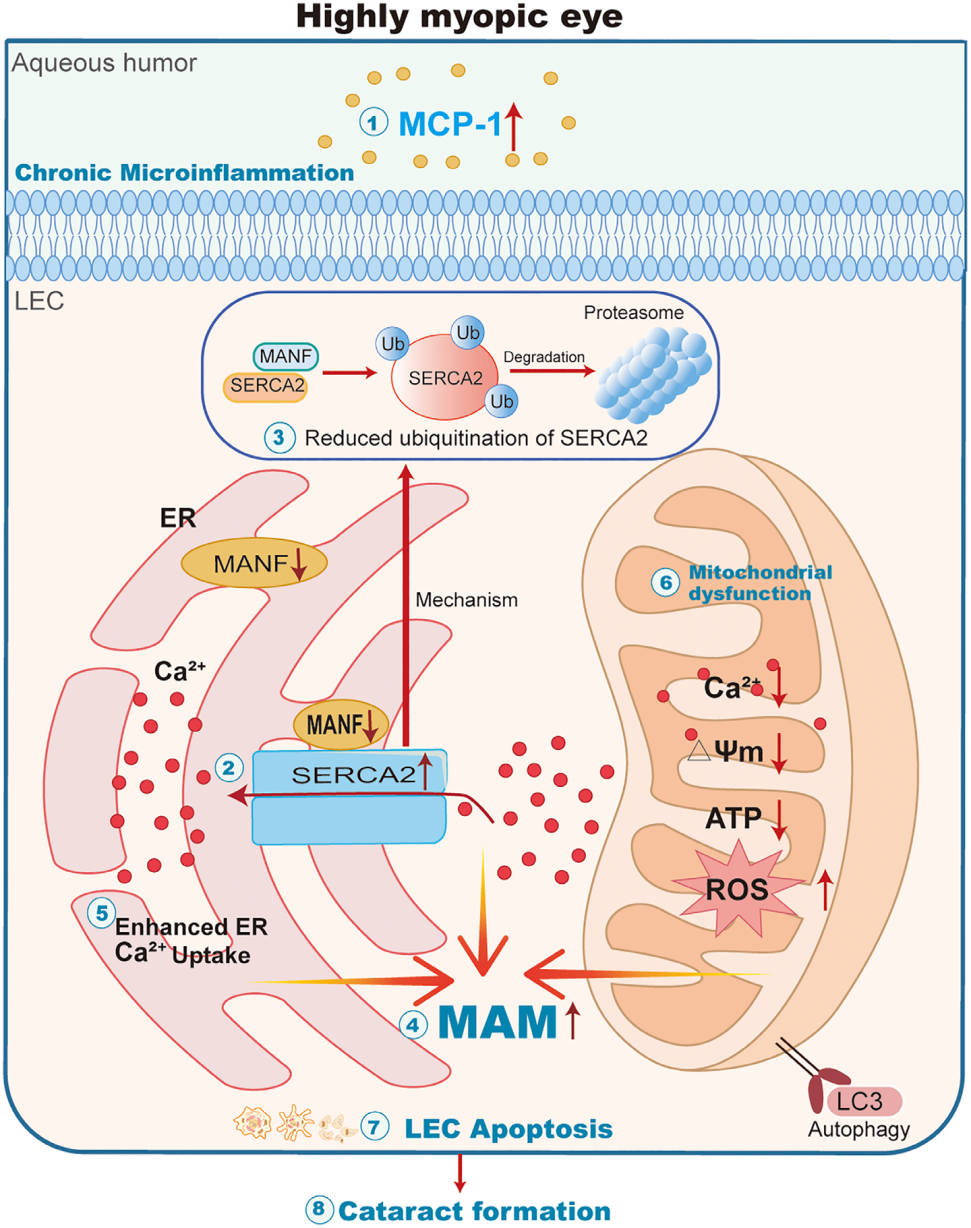
Schematic model of microinflammation‐driven MANF loss promoting cataractogenesis in high myopia via SERCA2‐dependent MAM disruption.

The chronic microinflammatory milieu in high myopia (HM), marked by elevated MCP‐1, suppresses MANF in the lens epithelial cells (LECs). Under physiological conditions, MANF acts as an intraluminal sensor at mitochondria‐associated ER membranes (MAMs), promoting the ubiquitin‐mediated degradation of endoplasmic reticulum (ER) Ca^2+^ pump SERCA2. However, microinflammation‐induced MANF depletion leads to pathological SERCA2 accumulation, triggering detrimental MAM hyperassembly. This causes ER Ca^2^^+^ overload, blunted cytosolic Ca^2^^+^ transients with prolonged decay, reduced ER‐to‐mitochondria Ca^2^^+^ transfer, and suppressed store‐operated Ca^2^^+^ entry (SOCE). Consequently, this disrupted Ca^2^^+^ homeostasis triggers a devastating cascade of cellular dysfunctions, including the collapse of mitochondrial membrane potential, profound bioenergetic failure, and exacerbated oxidative stress. These escalating insults overwhelm protective autophagy and commit LECs to apoptosis, thereby accelerating cataract formation in HM eyes.

## 4 Methods

### 4.1 Patient Enrollment and Sample Collection

This study was part of the Shanghai High Myopia Study (NCT03060585) and approved by the Ethics Committee of Eye & ENT Hospital, Fudan University (No. 2014055). All participants provided written informed consent. Consecutive patients undergoing cataract surgery between January and June 2023 were enrolled. HMC group (*n* = 50) had axial length ≥ 26.00 mm in both eyes and nuclear cataract (LOCS III NC > 3). ARC controls (*n* = 50) had axial length of 22.00–24.50 mm and cortical cataract (LOCS III C > 3). Exclusion criteria included ocular comorbidities (glaucoma, uveitis), previous ocular surgery or trauma, or systemic diseases such as diabetes or cancer.

During capsulorhexis in phacoemulsification, human lens epithelia (*n* = 100; 50 HMC/50 ARC) were collected and stored at –80 °C for further analyses. For molecular analyses (qRT‐PCR/Western blot), 3–6 pieces of lens epithelia were pooled per replicate. For immunofluorescence (IF), single epithelium was fixed in 4% PFA (4°C). For electron microscopy, single epithelium was fixed immediately at room temperature (2 h) and stored at 4 °C. All samples were processed within 24 h.

### 4.2 Aqueous Humor Collection and MCP‐1 ELISA

Aqueous humor (100 µL) was collected during phacoemulsification surgery via a paracentesis site before viscoelastic injection (DisCoVisc; Alcon). MCP‐1 levels were measured using the QuantiCyto Human MCP‐1 ELISA kit (High Sensitivity) according to the manufacturer's instructions.

### 4.3 Mouse Models

All animal procedures were approved by the Animal Ethics Committee of the Eye & ENT Hospital, Fudan University. Mice were housed under specific pathogen‐free conditions (22°C –24°C, 12‐h light/dark cycle) with free access to food and water. All in vivo experiments were conducted exclusively using male C57BL/6J mice.

#### 4.3.1 Lens‐Specific MANF Conditional Knockdown Mice

Conditional MANF knockdown mice (C57BL/6J background) were generated by GemPharmatech using the Cre‐LoxP system. MANF^flox/flox^; Prox1‐cre‐ERT2 (termed MANF cKD) and MANF^flox/flox^ mice (WT controls) received daily intraperitoneal tamoxifen (75 mg/kg in corn oil) for 7 days starting at 4 weeks of age to induce lens‐specific MANF deletion. At 8 weeks, MANF ablation was confirmed by western blot and immunofluorescence. MANF cKD mice developed progressive lens opacification and nuclear cataracts by 8 weeks.

#### 4.3.2 Defocus‐induced High Myopia Model

Five‐week‐old male mice were used. Refraction was measured in both eyes using an automated eccentric infrared photorefractor; animals with interocular difference >1 D were excluded. A −10 D lens was placed on the periorbital skin of the right eye for 4 weeks; lenses were checked and repositioned daily. Mice with a myopic shift ≥ 6 D in the right eye relative to the left were considered successful HM models. Mice with plano (0 D) lenses served as sham controls.

### 4.4 Cell Culture

Human LECs (SRA01/04 line) were maintained in DMEM supplemented with 10% FBS at 37 °C with 5% CO_2_. For microinflammation modeling, cells were treated with 100 ng/mL MCP‐1 for 48 h.

Primary mouse LECs were isolated from WT, MANF cKD, or HM mice and cultured in DMEM with 20% FBS, with medium refreshed every 48 h.

### 4.5 Transfections

LECs were transfected with pcDNA3.1‐MANF‐FLAG, MANF siRNA (siMANF‐1, siMANF‐2), MANF shRNA, SERCA2 siRNA (siSERCA2‐1, siSERCA2‐2, siSERCA2‐3) or corresponding negative controls (NC‐OE, siNC, shNC) using Lipofectamine 3000 according to the manufacturer's instructions. Sequences are provided in Table S1.

### 4.6 Slit‐Lamp Photography

Human and mouse lenses were imaged with a slit‐lamp camera under diffuse, slit, and retro‐illumination.

### 4.7 Lens Ex Vivo Culture

Mouse lenses were aseptically extracted, placed in high‐glucose DMEM containing 20% FBS, 100 U/mL penicillin and 100 µg/mL streptomycin, and maintained at 37°C with 5% CO_2_. Lens opacity was imaged against a black cross background using a stereomicroscope, and nuclear cataract area was quantified as the ratio of nuclear opacification area to total lens area using ImageJ.

### 4.8 Quantitative Real‐Time PCR (qRT‐PCR)

Total RNA was extracted, reverse transcribed using the HiFiScript gDNA removal cDNA Synthesis Kit, and subjected to qRT‐PCR with GAPDH as the endogenous control. Primer sequences are listed in Table S2.

### 4.9 Western Blotting

Protein lysates were prepared using RIPA buffer and quantified with a BCA assay. Equal amounts of protein (10 µg per lane) were separated on 10%–15% SDS‐PAGE gels and transferred onto PVDF membranes. After blocking, membranes were incubated overnight at 4°C with primary antibodies, followed by incubation with HRP‐conjugated secondary antibodies (1:5000) for 1 h at room temperature. Signals were developed using enhanced chemiluminescence. β‐actin or GAPDH was used as a loading control.

### 4.10 Antibodies

For western blotting, the following primary antibodies were used: anti‐ACSL4 (1:500, Immunoway, YT8070), anti‐AMPKα1 (1:1000, Proteintech, 10929‐2‐AP), anti‐ATF6 (1:1000, Abclonal, A25317), anti‐BAX (1:1000, Abclonal, A19684), anti‐BiP/GRP78 (1:1000, Cell Signaling Technology, 3177), anti‐cleaved caspase‐3 (1:500, Proteintech, 25128‐1‐AP), anti‐CHOP (1:2000, Abclonal), anti‐cytochrome c (1:1000, Proteintech, 10993‐1‐AP), anti‐GAPDH (1:50 000, Abclonal), anti‐IL‐1β (1:2000, Proteintech, 16806‐1‐AP), anti‐IP3R (1:1000, Abclonal, A4436), anti‐KDELR2 (1:1000, Proteintech, 17351‐1‐AP), anti‐LC3B (1:1000, Cell Signaling Technology, 3868), anti‐MANF (1:5000, Abcam, ab264402), anti‐MCU (1:1000, Cell Signaling Technology, 14997), anti‐MFN2 (1:5000, Proteintech, 12186‐1‐AP), anti‐NLRP3 (1:1000, Abcam, ab263899), anti‐NOX1 (1:500, Abclonal, A8527), anti‐NRF2 (1:1000, Abclonal, A21176), anti‐PERK (1:500, Proteintech, 24390‐1‐AP), anti‐phospho‐AMPKα1 (1:1000, Cell Signaling Technology, 2535S), anti‐phospho‐eIF2α (1:1000, Cell Signaling Technology, 3398), anti‐p62/SQSTM1 (1:20 000, Abclonal, A19700), anti‐SERCA2 (1:500, Abclonal, A0098), anti‐TOMM20 (1:1000, BD Biosciences, 612278), anti‐ubiquitin (1:1000, Proteintech, 10201‐2‐AP), and anti‐VDAC1 (1:5000, Abclonal, A19707). β‐Actin mouse mAb (1:2000, KIGENE, KI2654‐50) or rabbit mAb (1:2000, KIGENE, KWB040‐R) served as loading controls. HRP‐conjugated goat anti‐mouse (1:50 000, KIGENE, KI2663) and anti‐rabbit (1:50 000, KIGENE, KWB045) secondary antibodies were used for chemiluminescent detection. For immunofluorescence, Alexa Fluor 488/594‐conjugated secondary antibodies (Thermo Fisher, 1:500) were applied. All antibodies were validated for specificity in the respective applications.

### 4.11 Immunofluorescence and Confocal Microscopy

Cells or tissue sections were fixed in 4% paraformaldehyde, permeabilized with Triton X‐100, and blocked. For standard immunostaining, samples were incubated overnight at 4°C with primary antibodies (e.g., anti‐SERCA2), followed by appropriate fluorescent secondary antibodies (1:1000) for 1 h. Nuclei were counterstained with DAPI.

To precisely resolve the spatial relationship of MANF at mitochondria‐associated ER membranes (MAMs) and circumvent cross‐reactivity between same‐species primary antibodies (anti‐MANF, anti‐TOMM20, and anti‐Calnexin, all raised in rabbit), we employed a multiplexed labeling strategy using FlexAble 2.0 Antibody Labeling Kits (Proteintech, Rosemont, IL, USA). Prior to staining, purified primary antibodies were covalently conjugated to distinct fluorophores: rabbit anti‐MANF to CoraLite Plus 488 (Cat# KFA501), anti‐TOMM20 to CoraLite Plus 555 (Cat# KFA502), and anti‐Calnexin to CoraLite Plus 647 (Cat# KFA503). Two complementary sets of multiplexed staining were performed: (1) fixed cells co‐stained with DAPI, TOMM20‐555, Calnexin‐647, and MANF‐488; (2) live cells pre‐incubated with MitoTracker Red CMXRos (or MitoTracker Deep Red, 100 nM, 30 min) to label functional mitochondria, followed by fixation and co‐staining for Calnexin‐647 and MANF‐488.

High‐resolution Z‐stack images (≤0.5 µm intervals) for MAMs analysis were acquired using a Nikon Spatial Array Confocal (NSPARC) microscope (Equipment ID: CB1‐039‐05; Nikon Instruments, Tokyo, Japan) equipped with a 60**×**/1.4 NA oil‐immersion objective. Standard colocalization profiles were analyzed with Image‐Pro Plus software. To quantitatively evaluate asymmetric spatial overlap in multiplexed images, Manders' Colocalization Coefficients (M1 and M2) were calculated using the JACoP plugin in ImageJ/Fiji. For 3D visualization of mitochondria and the cytoskeleton, cells were stained with MitoTracker and phalloidin (actin), and Z‐stacks were acquired on a Nikon confocal microscope followed by 3D reconstruction using NIS‐Element Viewer 5.22 software.

### 4.12 TEM

Lens epithelia were spread on filter paper and treated with DMSO for 4 h, then fixed overnight in 2% paraformaldehyde/2.5% glutaraldehyde. Cultured HLECs were processed similarly following experimental treatments. Samples were washed, dehydrated, embedded in resin, and sectioned with a Leica EM UC7 ultramicrotome. Sections were examined under a Hitachi HT7800 electron microscope at 80 kV.

### 4.13 Subcellular Fractionation, MAM Isolation, and Proteinase K Protection Assay

MAMs, ER, cytosol, crude and purified mitochondria were isolated from SRA 01/04 LECs using differential centrifugation combined with a Percoll density gradient. Cells were trypsinized, pelleted, and homogenized in ice‐cold buffer (225 mm mannitol, 75 mm sucrose, 0.1 mm EGTA, 30 mm Tris‐HCl, pH *7.4*). Nuclei and debris were removed by low‐speed centrifugation (600 × g, 5 min, 4°C, twice). The supernatant was centrifuged (7000 × g, 10 min, 4°C) to pellet crude mitochondria, which were washed and resuspended in mannitol‐HEPES‐EGTA buffer.

For purification, the suspension was layered onto a 30% Percoll medium and ultracentrifuged (95 000 × g, 30 min, 4°C). The dense mitochondrial band and the diffuse MAM band were separately collected, diluted, and recovered by centrifugation. MAMs were pelleted at 100 000 × g for 1 h. Purity of fractions was confirmed by immunoblotting using established markers: ACSL4 (MAM), IP3R/SERCA2 (ER), cytochrome c (mitochondria), VDAC (MAM/mitochondria), and β‐actin (cytosol).

To determine the topological orientation of MANF, a Proteinase K protection assay was performed on the purified MAM fraction. The MAM fraction was equally divided into four aliquots: (1) untreated control; (2) treated with Proteinase K (50 µg/mL); (3) treated with Proteinase K and 1% Triton X‐100; and (4) treated with 1% Triton X‐100 alone. Samples were incubated on ice for 30 min, and the reaction was terminated by adding 5 mm phenylmethylsulfonyl fluoride (PMSF). Samples were subsequently boiled in SDS loading buffer to inactivate the protease and subjected to Western blot analysis.

### 4.14 Plasmid Construction and Mitochondria‐ER Contact Sites (MERCs) Visualization

A spGFP reporter was used to visualize MERCs^56^. Two lentiviral constructs were generated:

(1) **ER‐spGFP1‐10**: The spGFP1‐10 fragment, fused with a V5 tag and the C‐terminal ER transmembrane domain of human UBE2J2 (residues 228–259), was cloned into the PGMLV‐CMV‐MCS‐PGK‐Puro vector.

(2) **Mito‐2 × spGFP11**: The mitochondrial targeting sequence of human TOMM70 (residues 1–59), fused via a linker to two tandem spGFP11 fragments, was cloned into the PGMLV‐CMV‐MCS‐PGK‐Blasticidin vector.

SRA 01/ 04 human LECs and primary mouse LECs were co‐transfected with the two constructs. Stable polyclonal cell populations were established via dual antibiotic selection with puromycin (2 µg/mL) and blasticidin (10 µg/mL). Transfected cells were imaged using a Leica SP8 confocal microscope to visualize the reconstituted GFP signal at MERCs.

### 4.15 Intracellular Calcium Measurement

Cytosolic and mitochondrial Ca^2^^+^ dynamics were monitored using the fluorescent indicators Fluo‐4 AM and Rhod‐2 AM, respectively. In experiments measuring steady‐state intracellular calcium levels in differentiated LECs, the Calcium Quantification Kit – Red Fluorescence was used according to the manufacturer's instructions. Fluorescence was recorded in real‐time on a confocal microscope or quantified using a BioTek microplate reader (Ex/Em = 540/590 nm). Where indicated, cells were stimulated with ATP (100 µm) or treated with experimental compounds (e.g., CAP, 1–90 µm).

### 4.16 Real‐Time Spatiotemporal Calcium Dynamics Assays

To rigorously evaluate functional Ca^2+^ flux, multidimensional Ca^2+^ dynamics were continuously recorded at 37°C using a multimodal microplate reader (excitation/emission indicated below). For all dynamic assays, cells were maintained in buffered Normal HBSS (1.26 mm Ca^2+^, 0.49 mm Mg^2+^, 20 mm HEPES, pH *7.4*). For store‐operated Ca^2+^ entry (SOCE), a Ca^2+^‐free HBSS supplemented with 0.5 mm EGTA and 20 mm HEPES was utilized. 0.04% Pluronic F‐127 (Poloxamer 407) was used to facilitate AM‐ester dye loading. *(1) Cytosolic Ca^2+^ Transients and SERCA‐mediated Reuptake*: Cells were loaded with 2 µm Fluo‐4 AM (Ex/Em = 485/525 nm) for 40 min, washed, and de‐esterified for 20 min. Following a 1‐min baseline recording, 100 µm ATP was injected to trigger IP3‐mediated ER Ca^2+^ release. Cytosolic Ca^2^^+^ transient amplitude (Fluo 4 AM) upon ATP stimulation, calculated as ΔF = F_“peak” ‐F_“baseline”. The half‐life (*t*_1/2_) of the post‐peak fluorescence decay was calculated to reflect the SERCA‐driven Ca^2+^ reuptake rate. *(2) Mitochondrial Ca^2+^ Uptake*: Cells were loaded with 4 µm Rhod‐2 AM (Ex/Em = 549/578 nm) for 30 min. After de‐esterification, baseline mitochondrial Ca^2+^ was recorded, followed by ATP (100 µM) stimulation to evaluate ER‐to‐mitochondria Ca^2+^ transfer. Mitochondrial Ca^2^^+^ uptake amplitude (Rhod‐2 AM), reflecting ER‐mitochondria Ca^2^^+^ transfer efficiency at MAMs and calculated as Δ *F*_mito_ = *F*_peak, mito_  − *F*_baseline, mito_ *. (3) ER Resting Ca^2+^ Load*: Cells were incubated with 10 µm of the ER‐targeted probe Cal‐520ER AM (Ex/Em = 490/525 nm) supplemented with 1 mm Probenecid for 2.5 h at 37°C to ensure deep ER penetrance. Steady‐state baseline fluorescence directly representing the ER Ca^2+^ load was measured. Signal specificity was verified by the subsequent addition of 1 µm Thapsigargin (TG). *(4) Store‐Operated Ca^2+^ Entry (SOCE)*: Following Fluo‐4 AM loading, cells were rigorously washed and bathed in Ca^2+^‐free HBSS‐EGTA buffer. SERCA pumps were irreversibly inhibited by 1 µm TG to exhaust ER Ca^2+^ stores and initiate STIM1‐Orai1 coupling. After 10 min, 2 mm CaCl2 was acutely injected to trigger the massive influx of extracellular Ca^2+^ (SOCE amplitude was calculated using $\frac{F_{\mathrm{peak}}-F_{\mathrm{TG}\;\mathrm{bottom}}}{F_{0}}$ ).

### 4.17 Mitochondrial Function Analysis

Cellular mitochondrial integrity and function were assessed using the following fluorescent probes.

**MitoTracker Deep Red (100 nm, 30 min; Thermo Fisher)** visualized mitochondrial morphology and mass, exploiting its potential‐dependent accumulation in active mitochondria.

**MitoSOX Red (5 µm, 30 min; Invitrogen)** quantified mitochondrial superoxide by oxidation‐dependent red fluorescence (Ex/Em = 510/580 nm).

**JC‐1 (2 µm, 30 min; Sigma)** detected mitochondrial membrane potential (ΔΨm) shifts: healthy mitochondria (red J‐aggregates, Ex/Em = 585/590 nm) vs. depolarized (green monomers, Ex/Em = 514/529 nm).

**Rhodamine 123 (1 µm, 30 min; Abcam)** measured ΔΨm via potential‐dependent mitochondrial sequestration (green fluorescence, Ex/Em = 507/529 nm).

**Calcein‐AM (2 µm, 30 min; Sigma)** assessed cell viability, with intracellular esterases converting it to green‐fluorescent calcein (Ex/Em = 494/514 nm) in live cells.

### 4.18 Intracellular ATP Content

ATP concentration in HLECs and adipose tissue sections (iWAT, eWAT, BAT) was determined using the ATP Assay Kit (Abcam, AB83355) following the manufacturer's protocol.

### 4.19 Flow Cytometry Assays

#### 4.19.1 Apoptosis

Cells stained with FITC‐Annexin V/PI (BD Biosciences) for 15 min identified early (Annexin V^+^PI^−^) and late (Annexin V^+^PI^+^) apoptotic populations.

#### 4.19.2 ROS

DCFH‐DA (10 µm, 30 min; Sigma) detected intracellular ROS via oxidation to fluorescent DCF (Ex/Em = 488/525 nm).

#### 4.19.3 Cell Cycle

Ethanol‐fixed cells treated with PI/RNase A (BD Biosciences) were analyzed for DNA content to quantify G0/G1, S, and G2/M phases.

### 4.20 Mitophagy Detection

A dual‐fluorescence RFP‐GFP‐LC3 reporter (pGMLV‐CMV‐RFP‐GFP‐hLC3‐Puro) was transduced into SRA 01/04 human LECs or primary LECs (MOI = 20). After puromycin selection, mitochondrial localization was confirmed with MitoTracker Deep Red. Mitophagy was induced with 10 µm CCCP for 4 h. Yellow puncta (RFP+GFP+) indicated autophagosomes; red puncta (RFP+GFP‐) indicated acidified autolysosomes. Colocalization with mitochondria was analyzed by confocal microscopy.

### 4.21 High‐Throughput MANF Interactome Analysis

MANF‐interacting proteins were isolated from LEC lysates by IP with anti‐MANF antibody, followed by on‐bead trypsin digestion and LC‐MS/MS (Q Exactive HF‐X). Data were integrated with MAM proteomics using STRING network analysis.

### 4.22 Co‐IP

For Co‐IP analyses, clarified cell or tissue lysates were incubated with the specified primary antibody overnight at 4 °C. Protein A/G magnetic beads were then added, and the mixture was incubated for an additional 2 h at 4°C. Beads were washed extensively, and bound proteins were eluted for subsequent Western blot analysis.

### 4.23 GST (Glutathione S‐Transferase) Pull‐Down Assays

For GST pull‐down assays, an equal amount (0.5 mg) of GST‐tagged fusion protein and His6‐tagged fusion protein were mixed and incubated on ice for 3 h. Subsequently, the mixture was loaded onto Glutathione Sepharose 4B resin columns. After washing five times with wash buffer, proteins were eluted with wash buffer supplemented with 15 mm reduced glutathione. The eluates were separated using 12% SDS‐PAGE, transferred to PVDF membranes (Millipore, Billerica, MA, USA), and probed with anti‐His (Sigma‐Aldrich, Merck KGaA, Darmstadt, Germany). GST and His6 from Wuhan Genecreate (Wuhan, China) were used as negative controls. There were three replications for each pull‐down assay.

### 4.24 Ubiquitination Assay

To assess protein ubiquitination, LECs were co‐transfected with constructs expressing MANF and SERCA2. Following a 48 h transfection period, cells were treated with the proteasome inhibitor MG132 (10 µm) for 6 h to block degradation of ubiquitinated proteins. SERCA2 was then immunoprecipitated from the lysates using a specific antibody. Ubiquitin conjugates were detected by Western blotting using an anti‐ubiquitin antibody.

### 4.25 Protein Degradation (Cycloheximide Chase) Assay

Protein degradation kinetics were determined using a cycloheximide chase assay. SRA 01/04 LECs were treated with cycloheximide (100 µm) to inhibit new protein synthesis. Cells were harvested at the indicated time points (e.g., 0, 1, 2, 4, 8, 12 h), and total protein was extracted. SERCA2 protein levels were analyzed by Western blotting, and the half‐life was calculated by densitometric quantification of the bands over time.

### 4.26 Protein‐Protein Molecular Docking Analysis

The structural interaction between MANF and SERCA2b was predicted using the HDOCK server, which employs a hybrid docking strategy combining template‐based modeling and ab initio free docking. The protein sequences of mature human MANF (without signal peptide) and full‐length human SERCA2b were used as inputs. The generated binding models were ranked based on the knowledge‐driven iterative scoring function (ITScorePP). The top‐ranked configuration with the lowest docking score (–251.87) was selected for subsequent analysis. The three‐dimensional interaction interface, including hydrogen bonds and salt bridges, was visualized using PyMOL (Version 3.0.3), and two‐dimensional interaction maps were generated using LigPlot+.

### 4.27 Intravitreal Injection and AAV2‐Mediated MANF Overexpression

To restore MANF expression, recombinant adeno‐associated virus (AAV) was delivered via intravitreal injection. Mice were anesthetized, pupils were dilated, and a 33‐gauge blunt needle was inserted into the vitreous cavity at the pars plana. Under microscopic guidance, 1 µL of AAV suspension (100 ng virus) was injected. The overexpression vector AAV2‐CMV‐m‐Manf‐P2A‐EGFP (QianmoBio Co., Ltd, Shanghai, China) carried the mouse MANF coding sequence (NM_029103.4). Control mice received AAV2‐CMV‐EGFP.

### 4.28 Tissue Section and Histology

Mouse eyes were fixed, embedded in paraffin or OCT compound, and sectioned for immunofluorescence or histological staining.

### 4.29 Swept‐Source OCT

Anterior segment photography was conducted by slip lamp and whole‐eye imaging in mice were performed using a swept‐source OCT system (YG‐100K, TowardPi Medical Technology). Imaging was conducted after pupil dilation (0.5% tropicamide and 0.5% phenylephrine). Mice were anesthetized with 1% pentobarbital sodium prior to imaging.

### 4.30 Mitochondrial Respiration (Seahorse) Assay

Oxygen consumption rate was measured using the Seahorse XFe96 Analyzer. Cells were seeded in XFe96 plates, and mitochondrial stress test (oligomycin, FCCP, rotenone/antimycin A) was performed according to the manufacturer's protocol.

### 4.31 Statistical Analysis

Data are presented as mean ± standard deviation (SD). All experiments were independently repeated at least three times (biological replicates), with specific sample sizes (n) detailed in the respective figure legends. The Shapiro‐Wilk test was applied to assess the normality of data distribution. For comparisons between two groups, either the unpaired two‐tailed Student's t‐test (for parametric data) or the Mann‐Whitney U test (for non‐parametric data) was used, as appropriate. One‐way analysis of variance (ANOVA) test was used to compare the differences among multiple groups. A p value of < 0.05 was considered statistically significant.

## Author Contributions

X.L., H.L., Y.D., and X.Z. conceptualized and designed this study. X.L., H.L., and C.K. performed and analyzed the experiments. X.L., S.L. and C.K. performed and executed the bioinformatic analyses and supported the animal experiments. S.L., J.M., Z.H. and C.K. supported human sample collection and cell experiments. X.L., Y.D., and H.L. wrote the draft manuscript. Y.D. and X.Z. reviewed and edited the manuscript. Xin Liu, Hao Li, and Ching Kang contributed equally as co‐first authors; Yu Du and Xiangjia Zhu are co‐corresponding authors.All authors have read and agreed to the published version of the manuscript.

## Funding

This article was supported by research grants from the National Natural Science Foundation of China (82271069, 82371040, 81900839, 82122017, 81870642 and 81970780, 82501327), National Key Research and Development Program of China (2024YFC2510800), Science and Technology Innovation Action Plan of Shanghai Science and Technology Commission (23Y11909800), Outstanding Youth Medical Talents of Shanghai “Rising Stars of Medical Talents” Youth Development Program, Shanghai Municipal Health Commission Project (2024ZZ1025 and 20244Z0015), Clinical Research Plan of Shanghai Shenkang Hospital Development Center (SHDC12026129), and Hospital‐Local Government Cooperation Project of Xuhui District (25XHYD‐26).

## Ethics Statement

This study was part of the Shanghai High Myopia Study, and all human sample collection and experimental procedures were approved by the Ethics Committee of Eye & ENT Hospital, Fudan University (Approval No. NCT03060585). All animal care and experimental protocols were conducted in strict accordance with the ARVO Statement for the Use of Animals in Ophthalmic and Vision Research and were approved by the Institutional Animal Care and Use Committee (IACUC) of Fudan University (Approval No. 2014055).

## Consent

Written informed consent was obtained from all participants (or their legally authorized representatives) prior to enrollment. Patient data were de‐identified and handled in accordance with the Declaration of Helsinki.

## Conflicts of Interest

The authors declare no conflicts of interests.

## Supporting information




**Supporting File 1**: advs76342‐sup‐0001‐FigureS1‐S5.zip.

**Supporting File 2**: advs76342‐sup‐0002‐TableS1‐S2.docx.

## Data Availability

The data that support the findings of this study are available from the corresponding author upon reasonable request.

## References

[1] M. Nakanishi , “Cellular Senescence as a Source of Chronic Microinflammation That Promotes the Aging Process,” Proceedings of the Japan Academy, Series B 101 (2025): 224–237, 10.2183/pjab.101.014. PMC12321498 40222899

[2] X. Shen , M. Li , Y. Li , et al., “Bazi Bushen Ameliorates Age‐related Energy Metabolism Dysregulation by Targeting the IL‐17/TNF Inflammatory Pathway Associated With SASP,” Chinese Medicine 19 (2024): 61, 10.1186/s13020-024-00927-9. PMC11005220 38594761

[3] J. E. Anttila , O. S. Mattila , H.‐K. Liew , et al., “MANF Protein Expression Is Upregulated in Immune Cells in the Ischemic human Brain and Systemic Recombinant MANF Delivery in Rat Ischemic Stroke Model Demonstrates Anti‐inflammatory Effects,” Acta Neuropathologica Communications 12 (2024): 10, 10.1186/s40478-023-01701-y. PMC10792833 38229173

[4] L. Pu , J. Liu , S. Kong , et al., “Therapeutic Potential of PCSK9 Inhibitors in Regulating Neuroinflammation in Acute Ischemic Stroke,” CNS Drugs 40 (2026): 657–667, 10.1007/s40263-026-01278-9. PMC13095988 41760861

[5] X. Liu , Y. Ding , J. Fu , et al., “Nanomaterial‐induced Metal Ion Interference Therapy in Cancer Treatment: From Tumor Microenvironment Reprogramming to Cell Fate Regulating,” Colloids and Surfaces B: Biointerfaces 263 (2026): 115576, 10.1016/j.colsurfb.2026.115576. 41763116

[6] D. V. Ziegler , N. Martin , and D. Bernard , “Cellular Senescence Links Mitochondria‐ER Contacts and Aging,” Communications Biology 4 (2021): 1323, 10.1038/s42003-021-02840-5. PMC8613202 34819602

[7] A. A. Mohan and P. Talwar , “MAM Kinases: Physiological Roles, Related Diseases, and Therapeutic Perspectives—A Systematic Review,” Cellular & Molecular Biology Letters 30 (2025): 35, 10.1186/s11658-025-00714-w. PMC11951743 40148800

[8] L. Boyman , M. Karbowski , and W. J. Lederer , “Regulation of Mitochondrial ATP Production: Ca^2+^ Signaling and Quality Control,” Trends in Molecular Medicine 26 (2020): 21–39, 10.1016/j.molmed.2019.10.007. PMC7921598 31767352

[9] J. Wei , J. Liu , H. Wang , et al., “Nanoplastic Propels Diet‐induced NAFL to NASH via ER‐mitochondrial Tether‐controlled Redox Switch,” Journal of Hazardous Materials 465 (2024): 133142, 10.1016/j.jhazmat.2023.133142. 38061129

[10] Q. Perrier , V. Lisi , K. Fisherwellman , et al., “Therapeutic Transplantation of Mitochondria and Extracellular Vesicles: Mechanistic Insights Into Mitochondria Bioenergetics, Redox Signaling, and Organelle Dynamics in Preclinical Models,” Free Radical Biology and Medicine 238 (2025): 473–495, 10.1016/j.freeradbiomed.2025.06.040. PMC12382009 40570985

[11] S. Missiroli , S. Patergnani , N. Caroccia , et al., “Mitochondria‐Associated Membranes (MAMs) and Inflammation,” Cell Death & Disease 9 (2018): 329, 10.1038/s41419-017-0027-2. PMC5832426 29491386

[12] Y. Du , J. Meng , W. He , J. Qi , Y. Lu , and X. Zhu , “Complications of High Myopia: An Update From Clinical Manifestations to Underlying Mechanisms,” Advances in Ophthalmology Practice and Research 4 (2024): 156–163, 10.1016/j.aopr.2024.06.003. PMC11260019 39036706

[13] J. B. Jonas , R. A. Jonas , M. M. Bikbov , Y. X. Wang , and S. Panda‐Jonas , “Myopia: Histology, Clinical Features, and Potential Implications for the Etiology of Axial Elongation,” Progress in Retinal and Eye Research 96 (2023): 101156, 10.1016/j.preteyeres.2022.101156. 36585290

[14] B. A. Holden , T. R. Fricke , D. A. Wilson , et al., “Global Prevalence of Myopia and High Myopia and Temporal Trends From 2000 Through 2050,” Ophthalmology 123 (2016): 1036–1042, 10.1016/j.ophtha.2016.01.006. 26875007

[15] H. J. Yu , M. T. Kuo , and P. C. Wu , “Clinical Characteristics of Presenile Cataract: Change Over 10 Years in Southern Taiwan,” BioMed Research International 2021 (2021): 9385293, 10.1155/2021/9385293. PMC8016560 33834076

[16] X.‐J. Zhu , P. Zhou , K.‐K. Zhang , J. Yang , Y. Luo , and Y. Lu , “Epigenetic Regulation of αA‐crystallin in High Myopia‐induced Dark Nuclear Cataract,” PLoS One 8 (2013): 81900, 10.1371/journal.pone.0081900. PMC3849391 24312600

[17] A. G. Tan , A. Kifley , Y.‐C. Tham , et al., “Six‐Year Incidence of and Risk Factors for Cataract Surgery in a Multi‐ethnic Asian Population,” Ophthalmology 125 (2018): 1844–1853, 10.1016/j.ophtha.2018.07.026. 30077615

[18] X. Zhu , D. Li , Y. Du , W. He , and Y. Lu , “DNA Hypermethylation‐mediated Downregulation of Antioxidant Genes Contributes to the Early Onset of Cataracts in Highly Myopic Eyes,” Redox Biology 19 (2018): 179–189, 10.1016/j.redox.2018.08.012. PMC6122317 30172102

[19] X. Zhu , J. Meng , C. Han , et al., “CCL2‐mediated Inflammatory Pathogenesis Underlies High Myopia‐related Anxiety,” Cell Discovery 9 (2023): 94, 10.1038/s41421-023-00588-2. PMC10497683 37699875

[20] Q. Yu , C. Wang , Z. Liu , et al., “Association Between Inflammatory Cytokines and Oxidative Stress Levels in Aqueous Humor With Axial Length in human Myopia,” Experimental Eye Research 237 (2023): 109670, 10.1016/j.exer.2023.109670. 37806610

[21] X. Zhu , K. Zhang , W. He , et al., “Proinflammatory Status in the Aqueous Humor of High Myopic Cataract Eyes,” Experimental Eye Research 142 (2016): 13–18, 10.1016/j.exer.2015.03.017. 25805322

[22] J. Zhang , K. Kamoi , Y. Zong , M. Yang , Y. Zou , and K. Ohno‐Matsui , “Inflammation and Immune Pathways in Myopia: An Overview on Pathomechanisms and Treatment Prospects,” Clinical Reviews in Allergy & Immunology 68 (2025): 98, 10.1007/s12016-025-09094-7. PMC12589388 41191163

[23] S. Pan , J. Yuan , Y. Jin , et al., “Innate Immune Responsive Inflammation in Development of Progressive Myopia,” Eye 38 (2024): 1542–1548, 10.1038/s41433-024-02947-z. PMC11126664 38287111

[24] T. Yang , J. Qi , and H. Xu , “The Role of Inflammation in Myopic Retinopathy,” Frontiers in Ophthalmology 5 (2025): 1632047, 10.3389/fopht.2025.1632047. PMC12404955 40909333

[25] D. Guo , J. Qi , Y. Du , et al., “Tear Inflammatory Cytokines as Potential Biomarkers for Myopic Macular Degeneration,” Experimental Eye Research 235 (2023): 109648, 10.1016/j.exer.2023.109648. 37704045

[26] X. Zhu , Y. Du , D. Li , et al., “Aberrant TGF‐β1 Signaling Activation by MAF Underlies Pathological Lens Growth in High Myopia,” Nature Communications 12 (2021): 2102, 10.1038/s41467-021-22041-2. PMC8032689 33833231

[27] L. Wei , Y. Du , S. Gao , et al., “TGF‐β1‐induced m6A Modifications Accelerate Onset of Nuclear Cataract in High Myopia by Modulating the PCP Pathway,” Nature Communications 16 (2025): 3859, 10.1038/s41467-025-58995-w. PMC12022316 40274784

[28] D. Guo , Y. Du , X. Liu , D. Li , L. Wei , and X. Zhu , “Enhanced Ferroptosis Sensitivity Promotes the Formation of Highly Myopic Cataract via the DDR2‐Hippo Pathway,” Cell Death & Disease 16 (2025): 64, 10.1038/s41419-025-07384-8. PMC11790942 39900894

[29] Q. Wei , X. Zhuang , J. Fan , et al., “Proinflammatory and Angiogenesis‐related Cytokines in Vitreous Samples of Highly Myopic Patients,” Cytokine 137 (2021): 155308, 10.1016/j.cyto.2020.155308. 33128924

[30] L. Jian , Z. Huang , Y. Du , and X. Zhu , “High Myopia as a Risk Factor for Severe Liver Disease in Individuals With Liver Dysfunction: Evidence From a Prospective Cohort,” Journal of Clinical Medicine 14 (2025): 5860, 10.3390/jcm14165860. PMC12387578 40869686

[31] S. Gao , T. Chen , C. Deng , G. Liu , and Z. Wei , “An Endoplasmic Reticulum Stress‐responsive Nanocomposite Hydrogel for Diabetic Wound Healing Through a Fibroblast‐immune Cell Dual Regulation Hub,” Journal of Nanobiotechnology 23 (2025): 689, 10.1186/s12951-025-03732-0. PMC12551225 41137149

[32] J. Huang , W. Yu , Q. He , et al., “Autophagy Facilitates Age‐Related Cell Apoptosis—A New Insight From Senile Cataract,” Cell Death & Disease 13 (2022): 37, 10.1038/s41419-021-04489-8. PMC8748728 35013122

[33] H. Zuo , X. Liu , B. Lv , et al., “Autophagy‐induced NR2F1 Activation Promotes the Apoptosis of Lens Epithelial Cells and Facilitates Cataract‐associated Fibrosis Through Targeting STAT3,” Genes & Diseases 12 (2025): 101549, 10.1016/j.gendis.2025.101549. PMC12242405 40641526

[34] S. Liu , Y. Wang , Y. Zhang , X. Wang , and L. Wang , “Mesencephalic Astrocyte‐Derived Neurotrophic Factor (MANF) Mitigates Neuroinflammation and Cognitive Impairment by Modulating Glial Activation in Sepsis‐Associated Encephalopathy,” Neurochemical Research 50 (2024): 39, 10.1007/s11064-024-04296-5. 39612058

[35] P. Dai , P. Wang , X. Chen , et al., “Mesencephalic Astrocyte‐Derived Neurotrophic Factor (MANF) Restricts Inflammatory Progression Through Limiting Macrophage Infiltration in DRG and Sciatic Nerve During Diabetic Peripheral Neuropathy,” ACS Chemical Neuroscience 16 (2025): 945–959, 10.1021/acschemneuro.5c00021. 39970444

[36] K.‐G. Zhou , Y.‐B. Huang , Z.‐W. Zhu , et al., “Mesencephalic Astrocyte‐Derived Neurotrophic Factor Inhibits Neuroinflammation Through Autophagy‐Mediated α‐Synuclein Degradation,” Archives of Gerontology and Geriatrics 131 (2025): 105738, 10.1016/j.archger.2024.105738. 39761611

[37] H. Xie , P. Zhang , S. Yang , et al., “Myeloid‐Derived MANF Ameliorates Ethanol‐Induced Liver Injury by Enhancing microRNA‐223 Expression,” Journal of Gastroenterology 60 (2025): 877–890, 10.1007/s00535-025-02240-0. 40111540

[38] H. Wu , H. Li , W. Wen , et al., “MANF Protects Pancreatic Acinar Cells Against Alcohol‐Induced Endoplasmic Reticulum Stress and Cellular Injury,” Journal of Hepato‐Biliary‐Pancreatic Sciences 28 (2021): 883–892, 10.1002/jhbp.928. PMC8397795 33644980

[39] C. Wang , Q. Bao , C. Hou , et al., “Mono‐Macrophage‐Derived MANF Alleviates Bacterial Myocarditis by Inhibiting NF‐kappaB Activation and Myocardial Inflammation,” Inflammation 44 (2021): 1916–1926, 10.1007/s10753-021-01469-0. 33939070

[40] R. Lindström , P. Lindholm , M. Palgi , M. Saarma , and T. I. Heino , “In Vivo Screening Reveals Interactions Between Drosophila Manf and Genes Involved in the Mitochondria and the Ubiquinone Synthesis Pathway,” BMC Genetics 18 (2017): 52, 10.1186/s12863-017-0509-3. PMC5455201 28578657

[41] Y. Shiga , A. G. Rangel Olguin , S. El Hajji , et al., “Endoplasmic Reticulum Stress‐related Deficits in Calcium Clearance Promote Neuronal Dysfunction That Is Prevented by SERCA2 Gene Augmentation,” Cell Reports Medicine 5 (2024): 101839, 10.1016/j.xcrm.2024.101839. PMC11722116 39615485

[42] C. Ren , C. Hu , M. Hu , Y. Wu , Y. Yang , and F. Lu , “Melatonin Protects RPE Cells From Necroptosis and NLRP3 Activation via Promoting SERCA2‐Related Intracellular Ca^2+^ Homeostasis,” Phytomedicine 135 (2024): 156088, 10.1016/j.phymed.2024.156088. 39341129

[43] Y. Nakada , A. S. Titus , W. Mizushima , et al., “p22phox Prevents the Oxidation of SERCA2a and Stabilizes it in the Heart,” Nature Cardiovascular Research 4 (2025): 1187–1205, 10.1038/s44161-025-00699-x. PMC12436179 40903547

[44] P. A. Bidwell , S. L. Yuen , J. Li , et al., “A Large‐Scale High‐Throughput Screen for Modulators of SERCA Activity,” Biomolecules 12 (2022): 1789, 10.3390/biom12121789. PMC9776381 36551215

[45] B. Cai , M. Ma , J. Zhang , et al., “LncEDCH1 Improves Mitochondrial Function to Reduce Muscle Atrophy by Interacting With SERCA2,” Molecular Therapy Nucleic Acids 27 (2022): 319–334, 10.1016/j.omtn.2021.12.004. PMC8717430 35024244

[46] R. A. Kitamura , D. Hummel , A. Ustione , D. W. Piston , and F. Urano , “Dual Role of Neuroplastin in Pancreatic β Cells: Regulating Insulin Secretion and Promoting Islet Inflammation,” Proceedings of the National Academy of Sciences 121 (2024): 2411234121, 10.1073/pnas.2411234121. PMC11331099 39666939

[47] J. Cen , D. Zhao , X. Shi , et al., “N6‐methyladenosine‐mediated Upregulation of MANF Promotes ER Stress Resistance in Renal Cell Carcinoma,” Cell Death & Disease 16 (2025): 486, 10.1038/s41419-025-07798-4. PMC12222710 40603319

[48] R. Bhattacharyya , S. E. Black , M. S. Lotlikar , et al., “Axonal Generation of Amyloid‐β From Palmitoylated APP in Mitochondria‐associated Endoplasmic Reticulum Membranes,” Cell Reports 35 (2021): 109134, 10.1016/j.celrep.2021.109134. PMC8287518 34010653

[49] Y. Hu , H. Chen , L. Zhang , et al., “The AMPK‐MFN2 Axis Regulates MAM Dynamics and Autophagy Induced by Energy Stresses,” Autophagy 17 (2021): 1142–1156, 10.1080/15548627.2020.1749490. PMC8143230 32249716

[50] D. Naon , M. Zaninello , M. Giacomello , et al., “Critical Reappraisal Confirms that Mitofusin 2 is an Endoplasmic Reticulum–Mitochondria Tether,” Proceedings of the National Academy of Sciences 113 (2016): 11249–11254, 10.1073/pnas.1606786113. PMC5056088 27647893

[51] J. C. Christianson and P. Carvalho , “Order Through Destruction: How ER‐Associated Protein Degradation Contributes to Organelle Homeostasis,” The EMBO Journal 41 (2022): 109845, 10.15252/embj.2021109845. PMC8922271 35170763

[52] X. Wu and T. A. Rapoport , “Mechanistic Insights Into ER‐associated Protein Degradation,” Current Opinion in Cell Biology 53 (2018): 22–28, 10.1016/j.ceb.2018.04.004. PMC6131047 29719269

[53] S. Schoebel , W. Mi , A. Stein , et al., “Cryo‐EM Structure of the Protein‐conducting ERAD Channel Hrd1 in Complex With Hrd3,” Nature 548 (2017): 352–355, 10.1038/nature23314. PMC5736104 28682307

[54] M. Toth , S. Alhabib , B. Manoury , R. Bobe , and V. Leblais , “SERCA3, Ubiquitous but Specific Calcium Pumps?,” Cell Calcium 132 (2025): 103079, 10.1016/j.ceca.2025.103079. 40961904

[55] J. Viskupicova and L. M. Espinoza‐Fonseca , “Allosteric Modulation of SERCA Pumps in Health and Disease: Structural Dynamics, Posttranslational Modifications, and Therapeutic Potential,” Journal of Molecular Biology 437 (2025): 169200, 10.1016/j.jmb.2025.169200. PMC12353849 40349954

[56] H.‐X. Chen , X.‐C. Wang , H.‐T. Hou , et al., “Lysine Crotonylation of SERCA2a Correlates to Cardiac Dysfunction and Arrhythmia in Sirt1 Cardiac‐specific Knockout Mice,” International Journal of Biological Macromolecules 242 (2023): 125151, 10.1016/j.ijbiomac.2023.125151. 37270127

[57] W. Guo , W. Guo , B. Chen , et al., “NEXN Protects Against Vascular Calcification by Promoting SERCA2 SUMOylation and Stabilization,” Nature Communications 16 (2025): 8074, 10.1038/s41467-025-63462-7. PMC12397270 40883305

[58] W. Zhang , M. Zhang , Z. Sha , et al., “TRIB2 promotes Pulmonary Artery Smooth Muscle Cell Proliferation Through SERCA2 Ubiquitination in Pulmonary Hypertension,” Free Radical Biology and Medicine 243 (2025): 414–433, 10.1016/j.freeradbiomed.2025.11.009. 41213438

[59] J. B. Parys and F. O. Lemos , “The Interplay Between Associated Proteins, Redox State and Ca^2+^ in the Intraluminal ER Compartment Regulates the IP3 Receptor,” Cell Calcium 117 (2024): 102823, 10.1016/j.ceca.2023.102823. 37976974

[60] M. Meng , Y. Jiang , Y. Wang , et al., “β‐Carotene Targets IP3R/GRP75/VDAC1‐MCU Axis to Renovate LPS‐induced Mitochondrial Oxidative Damage by Regulating STIM1,” Free Radic Biol Med 201 (2023): 1–14. 10.1016/j.freeradbiomed.2023.05.021 37270031

[61] S. Pi , W. Liu , J. Zhao , et al., “SERCA2 Dysfunction Drives Vascular Calcification via Coupling With TSPO‐MCU at Mitochondria‐Associated Endoplasmic Reticulum Membranes,” Pharmacological Research 227 (2025): 107558. 10.1016/j.phrs.2026.108177 41932666

[62] R. Zhang , W. Li , J. Wang , et al., “Cell‐penetrating Peptide‐functionalized Biomimetic Nanovesicles for Efficient Cataract Treatment via Enhanced Corneal Penetration and Lens‐mitochondria Dual Targeting,” Bioactive Materials 57 (2026): 305–322. 10.1016/j.bioactmat.2025.11.016 PMC12661988 41323208

[63] Z. Guo , X. Ma , R. X. Zhang , and H. Yan , “Oxidative Stress, Epigenetic Regulation and Pathological Processes of Lens Epithelial Cells Underlying Diabetic Cataract,” Advances in Ophthalmology Practice and Research 3 (2023): 180–186, 10.1016/j.aopr.2023.10.001. PMC10724013 38106550

[64] M. Hashemi , P. Shafiei Asheghabadi , M. Moassesfar , et al., “Exploiting Autophagy and Related Pathways: Pioneering New Horizons in Cataract Therapy,” Apoptosis 30 (2025): 1931–1960, 10.1007/s10495-025-02134-9. 40634815

[65] Z. Yang , X. Zhao , J. Xu , W. Shang , and C. Tong , “A Novel Fluorescent Reporter Detects Plastic Remodeling of Mitochondria–ER Contact Sites,” Journal of Cell Science 131 (2018): jcs208686, 10.1242/jcs.208686. 29158224

