# Supplementary figures and images for: MAM‐Localized MANF Counteracts Microinflammatory Stress to Attenuate Mitochondrial Dysfunction and Cataractogenesis in High Myopia
Source: Adv Sci (Weinh). 2026 Jul 8:e76342. Online ahead of print. doi: 10.1002/advs.76342 (PMC13345693; doi:10.1002/advs.76342)

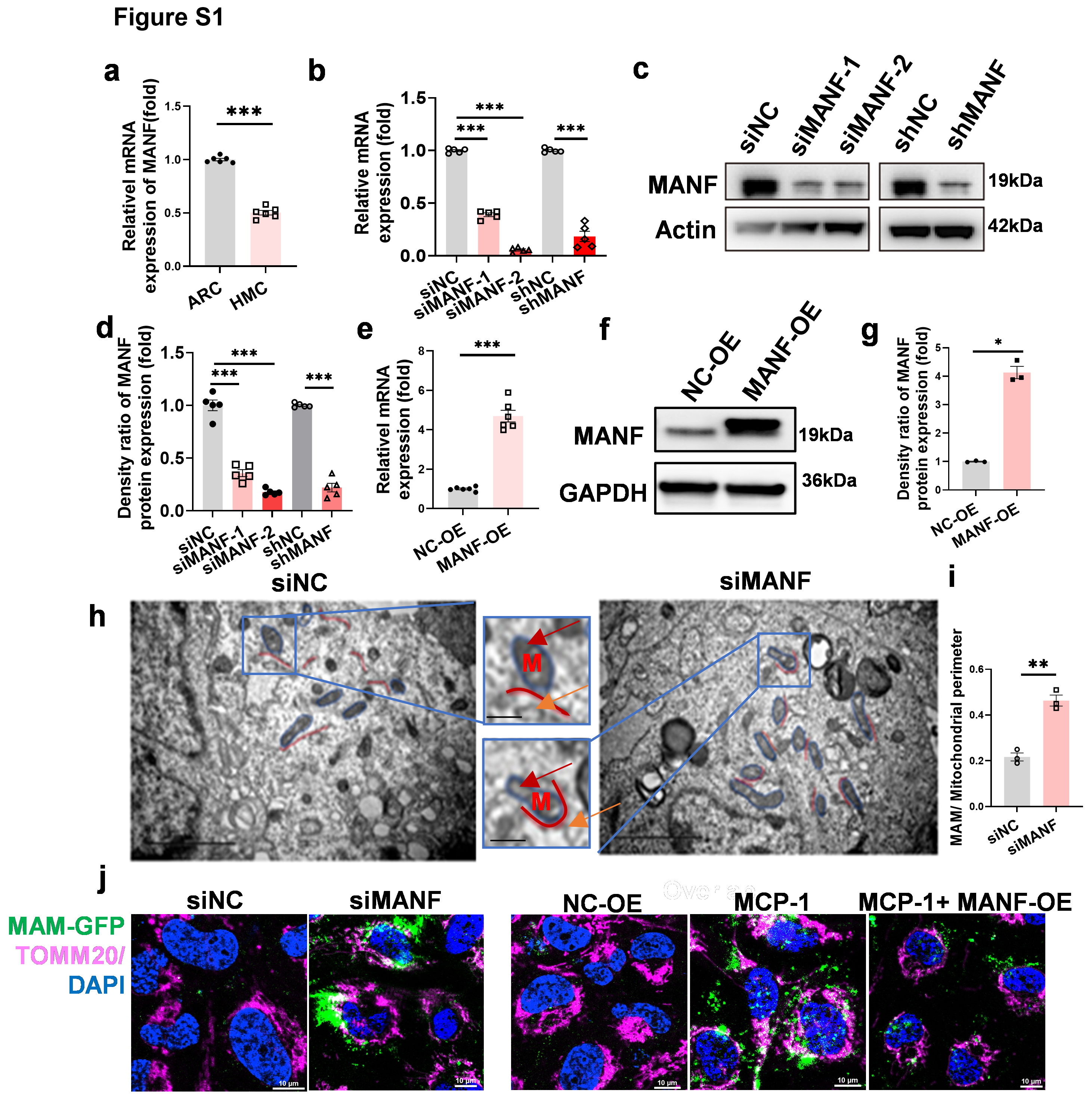

Supplement: Supplementary file 1 — Supporting File 1: advs76342‐sup‐0001‐FigureS1‐S5.zip. [file ADVS-9999-e76342-s001.zip › Figure S1.tif]

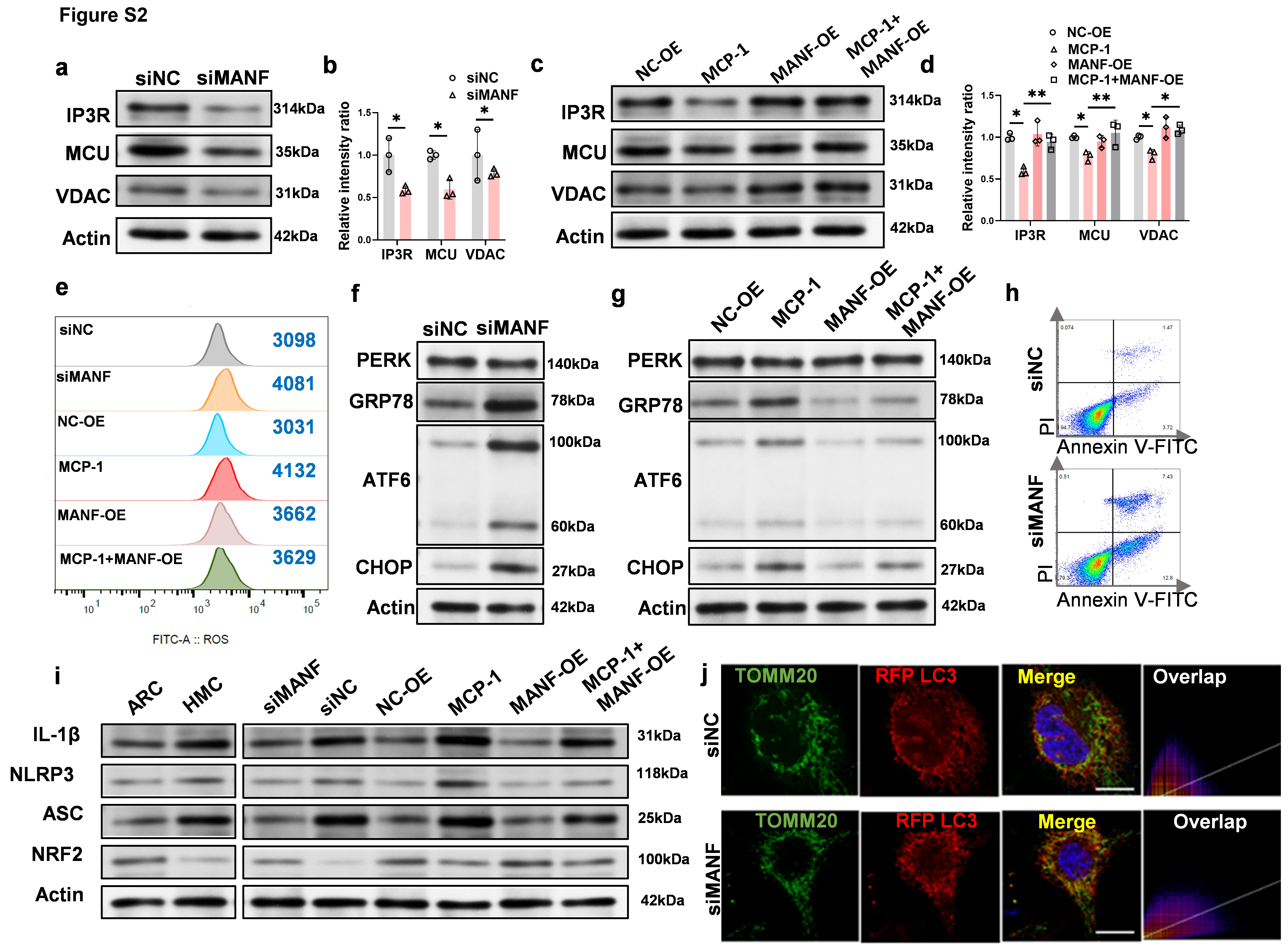

Supplement: Supplementary file 1 — Supporting File 1: advs76342‐sup‐0001‐FigureS1‐S5.zip. [file ADVS-9999-e76342-s001.zip › Figure S2.tif]

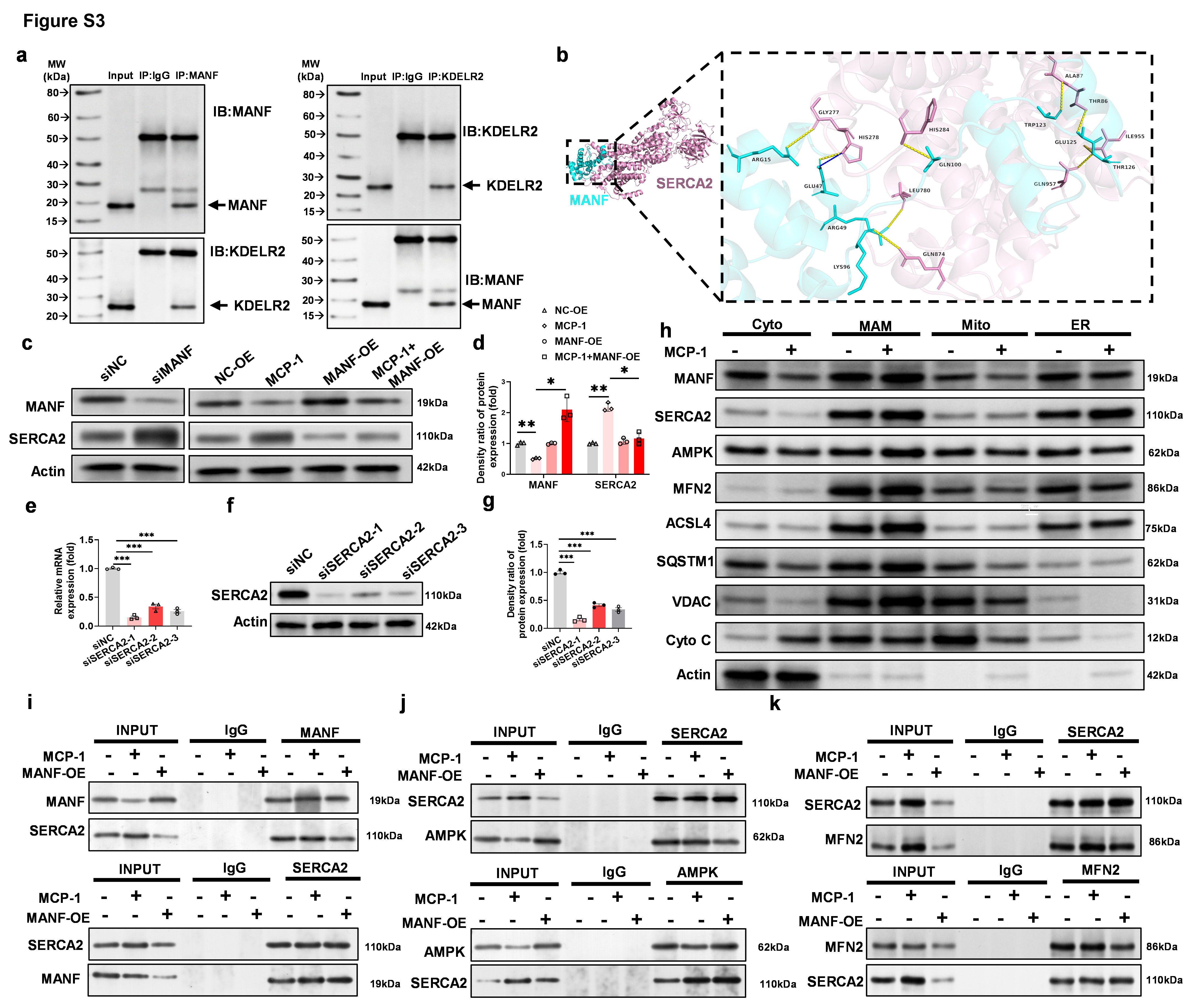

Supplement: Supplementary file 1 — Supporting File 1: advs76342‐sup‐0001‐FigureS1‐S5.zip. [file ADVS-9999-e76342-s001.zip › Figure S3-R1.tif]

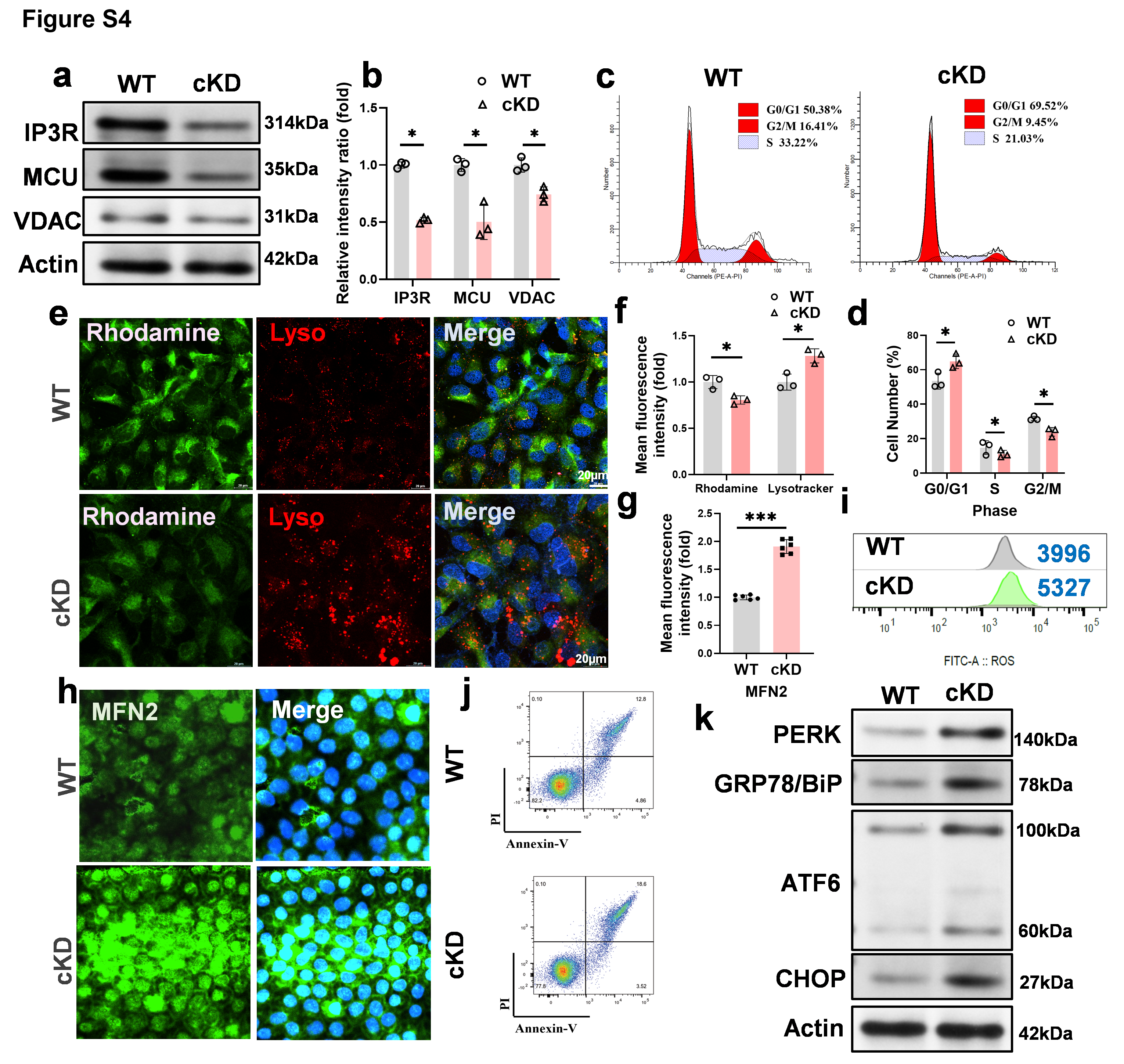

Supplement: Supplementary file 1 — Supporting File 1: advs76342‐sup‐0001‐FigureS1‐S5.zip. [file ADVS-9999-e76342-s001.zip › Figure S4.tif]

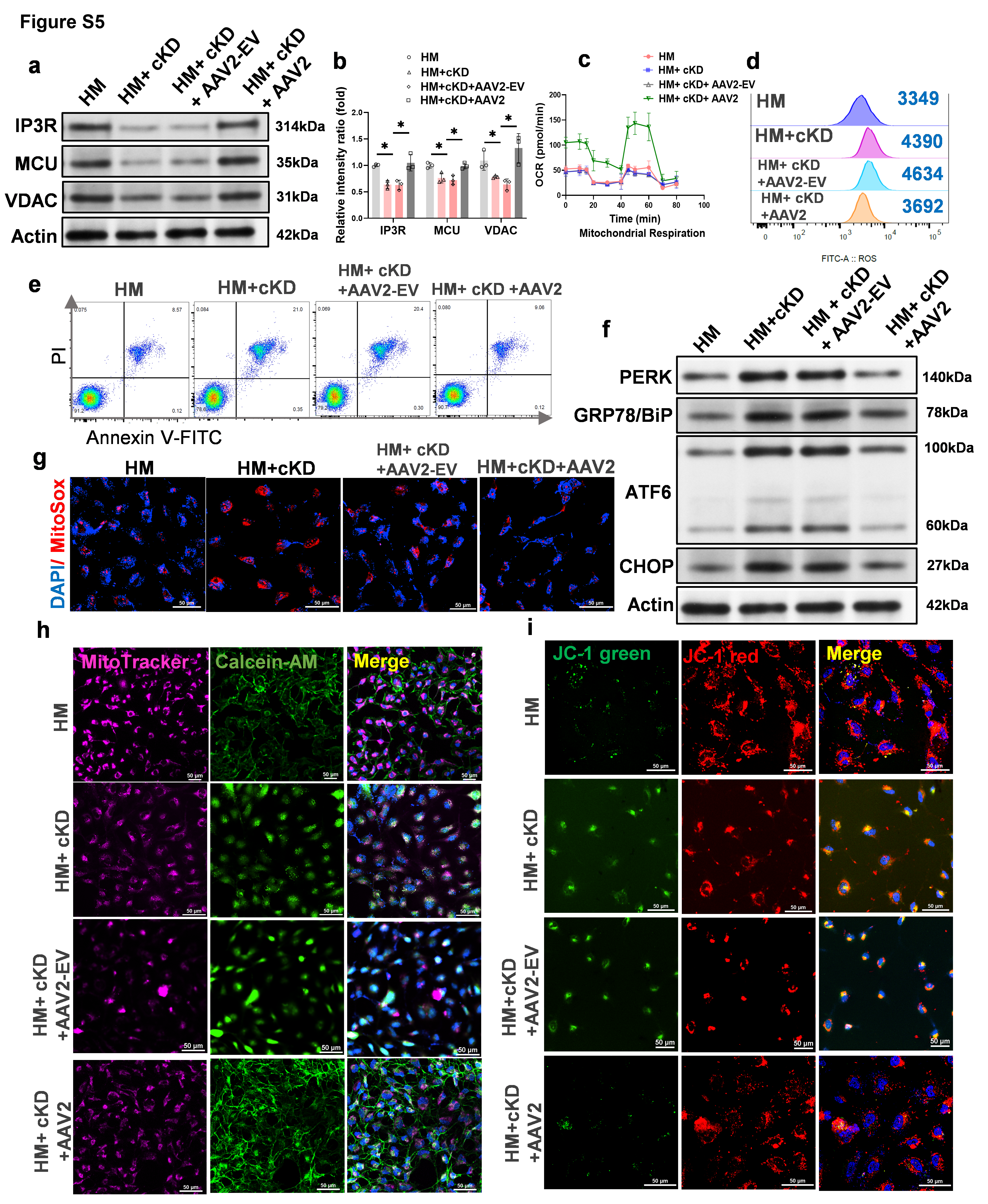

Supplement: Supplementary file 1 — Supporting File 1: advs76342‐sup‐0001‐FigureS1‐S5.zip. [file ADVS-9999-e76342-s001.zip › Figure S5.tif]
