# Supplementary material for: MAM‐Localized MANF Counteracts Microinflammatory Stress to Attenuate Mitochondrial Dysfunction and Cataractogenesis in High Myopia
Source: Adv Sci (Weinh). 2026 Jul 8:e76342. Online ahead of print. doi: 10.1002/advs.76342 (PMC13345693; doi:10.1002/advs.76342)
Supplement: Supplementary file 2 — Supporting File 2: advs76342‐sup‐0002‐TableS1‐S2.docx. [file ADVS-9999-e76342-s002.docx]

Supplementary Table 1 Sequence for siRNA

|  | Position | SS Sequence | AS Sequence |
| --- | --- | --- | --- |
| siMANF‑1 | 590 | CGAUUUGUAGUCUGCUCAAUC | UUGAGCAGACUACAAAUCGGU |
| siMANF‑2 | 446 | GAAGAAGCUCCGAGUUAAAGA | UUUAACUCGGAGCUUCUUCAG |
| Si-SERCA2-1 | 1097 | GAAAGUCAAUGUCGGUUUATT | UAAACCGACAUUGACUUUCTT |
| Si-SERCA2-2 | 1240 | GGAGUCAAACAGAAGAUCATT | UGAUCUUCUGUUUGACUCCTT |
| Si-SERCA2-3 | 213 | CCCACGAGCUGUCAACCAATT | UUGGUUGACAGCUCGUGGGTT |

Supplementary Table 2 Sequence of primers used to amplify target genes

| Primer | Primer sequence (5’-3’) |
| --- | --- |
| MANF  (Homo sapiens) | F: TTTACCAGGACCTCAAAGACAGA |
|  | R: TTGCTTCCCGGCAGAACTTTA |
| MANF  (Mus musculus) | F: TCTGGGACGATTTTACCAGGA |
|  | R: CTTGCTTCACGGCAAAACTTT |
| SERCA2  (Homo sapiens) | F: ATGGGGCTCCAACGAGTTAC |
|  | R: TTTCCTGCCATACACCCACAA |
| SERCA2  (Mus musculus) | F: GAGAACGCTCACACAAAGACC |
|  | R: ACTGCTCAATCACAAGTTCCAG |
| GAPDH  (Homo sapiens) | F: CAATGACCCCTTCATTGACC |
|  | R: GACAAGCTTCCCGTTCTCAG |
| GAPDH  (Mus musculus) | F: AGGTGCGGTGTGAACGGATTTG |
|  | R: TGAGTACCATGTAGTTGAGGTCA |
